# Deposition of Occupational Aerosol Particles in a Three-Dimensional Adult Nasal Cavity Model: An Experimental Study

**DOI:** 10.3390/bioengineering13020132

**Published:** 2026-01-23

**Authors:** Anna Rapiejko, Tomasz R. Sosnowski, Krzysztof Sosnowski, Dariusz Jurkiewicz

**Affiliations:** 1 Department of Otolaryngology and Laryngological Oncology with Clinical Department of Cranio-Maxillofacial Surgery, Military Institute of Medicine—National Research Institute, 04-141 Warsaw, Poland; 2 Faculty of Chemical and Process Engineering, Warsaw University of Technology, 00-645 Warsaw, Poland; 3 Institute of Control and Industrial Electronics, Faculty of Electrical Engineering, Warsaw University of Technology, 00-662 Warsaw, Poland

**Keywords:** nasal deposition, occupational aerosol particles, adult nasal cavity model

## Abstract

**Background**: Occupational exposure to aerosol particles can pose a substantial health risk. The study aimed to characterise the deposition of occupationally relevant aerosols in a 3D anatomical adult nasal cavity model under steady and unsteady flows. **Materials**: The deposition of aerosolised wheat flour, pine wood sanding dust, carbon black, and Arizona Test Dust A3 was quantified under steady flows (5, 7.5, and 20 L/min per nostril) and an unsteady breathing pattern generated by the commercial breathing simulator. Image analysis with custom software quantified the area covered by deposited particles. The Downstream Penetration Index (DPI) was determined from the outlet mass. **Results**: The highest segmental deposition occurred in the anterior segment of the lateral wall (WA) and septum (SA), with moderate values in the middle lateral wall (WM) and the lowest in the posterior lateral wall (WP, nasopharynx) and septum (SP). Arizona Test Dust A3 and carbon black demonstrated higher middle-posterior deposition and DPI, consistent with finer particle size distributions (PSD) and greater sub-10 µm fractions. In contrast, wheat flour and pine wood dust, with larger median particle sizes and lower sub-10 µm fractions, showed stronger anterior filtration and lower DPI. Increased flow enhanced anterior filtration of coarse particles and shifted deposition forward, aligning with increased inertial impaction, but elevated DPI for fine particles. Under unsteady flow, deposition was intermediate between 7.5 and 20 L/min. **Conclusions**: This study shows that PSD, morphology, and flow conditions influence nasal deposition. Coarse aerosols were filtered in the anterior nose, while fine-rich aerosols showed relatively greater middle-posterior deposition and higher DPI. These findings are essential for assessing occupational exposure and developing interventions and prevention strategies.

## 1. Introduction

The nasal cavities function as the primary entry point for airborne particles from the environment and the initial anatomical filter. Particle transport and deposition within the respiratory system depend on the particle size, shape, density, and airflow conditions [1]. Particle properties, exposure level, deposition sites, residence time in the respiratory tract, and host-specific risk factors influence interactions with respiratory cells. These factors contribute to the associated health risks.

Exposure to occupationally relevant aerosols is associated with a range of respiratory diseases that may affect various regions of the respiratory tract, with the nasal mucosa being the initial site of impact. The workplace environment can induce or exacerbate various forms of rhinitis, referred to as work-related rhinitis (WRR), which may involve both immunological and non-immunological mechanisms. WRR includes two main categories: occupational rhinitis (OR) and work-exacerbated rhinitis [2]. OR is defined as an inflammatory nasal disease with symptoms such as nasal congestion, sneezing, rhinorrhea, and itching, occurring in response to workplace exposures and not related to environmental factors outside the workplace. In contrast, work-exacerbated rhinitis refers to cases in which workplace exposures aggravate pre-existing or concurrent rhinitis [2].

The prevalence of occupational rhinitis is unclear [3]. In a review of nearly 60 occupations, Siracusa et al. found that OR is three times more frequent than occupational asthma (OA) [4]. This condition develops in a variety of work environments due to exposure to both high-molecular-weight (HMW) and low-molecular-weight (LMW) agents, with a higher prevalence associated with HMW agent exposure [5]. HMW agents (larger than 10 kDa) can mainly trigger IgE-mediated reactions. In contrast, LMW agents (smaller than 10 kDa) induce rhinitis symptoms mainly through non-IgE-mediated mechanisms, often acting as haptens [3].

Plant glycoproteins are common HMW sensitisers, and flour dust is the most prevalent cause of occupational respiratory allergy among workers in mills, bakeries, confectioneries, and grain processing facilities [6]. An analysis of occupational short-latency respiratory disease cases across various occupational groups reported to the UK-based Surveillance of Work-related and Occupational Respiratory Disease scheme from 1999 to 2019 indicated that bakers and flour confectioners had the highest incidence of rhinitis, with a significantly higher risk of rhinitis than of asthma [7]. Wheat, the primary cereal grain used in the baking industry, has a high allergenic potential. Wheat flour consists of starch and four main protein groups: glutenins, gliadins, globulins, and salt- or water-soluble albumins; among these, the latter two have the highest allergic potency in bakers [6,8]. Furthermore, flour dust may also contain other allergenic components, such as enzymes, additives, and microorganisms [9]. In the Kirkeleit et al. study, the extra-thoracic flour dust fraction, most relevant to upper respiratory tract flour-induced sensitisation, accounted for about 85% of measured inhalable flour dust [10,11].

Wood dusts are LMW agents that may cause harmful health effects, including in the upper respiratory tract. Occupational exposure occurs primarily in sawmills, furniture manufacturing, and the wood-based panel industry. Mechanical processing of dry wood produces the most dust, especially during sanding, sawing, and drilling [12]. Wood dust comes from dozens of tree species, including gymnosperms (conifers, such as pine, called softwoods) and angiosperms (deciduous trees, such as oak, called hardwoods). Wood dust mainly contains cellulose, hemicellulose, lignin, and many complex organic compounds. They differ in chemical properties due to the presence of different organic substances, such as essential oils, glucose, mannose, and galactose. The toxic effects of wood dust are usually due to biologically active chemical compounds, including terpenes, phenolic compounds, and flavonoids [12]. Wood dusts also vary in physical properties, including hardness, specific gravity, and particle size and shape. The majority of wood dust released during processing consists of extra-thoracic fraction particles. Upon deposition in the nasal cavities, these particles may induce both allergic and non-allergic occupational rhinitis, also referred to as irritant-induced rhinitis [13,14]. Wood dust is recognised as a carcinogenic substance [15]. Workers exposed to it have an increased risk of developing two histological types of malignant tumours in the nose and paranasal sinuses: adenocarcinoma and squamous cell carcinoma [16,17,18].

Carbon black is another factor that may influence health outcomes from occupational exposure, primarily through mechanisms dependent on inflammatory pathways and oxidative stress [19,20]. It is a quasi-graphitic form of nearly pure elemental carbon (>95%) that contains low levels of inorganic substances and extractable organics, including polycyclic aromatic hydrocarbons and nitro derivatives of aromatic hydrocarbons [20,21]. Carbon black particles form grape-like aggregates of fused spherical primary particles, which are further grouped into larger agglomerates [21]. It is produced by partial combustion or thermal decomposition of hydrocarbons under controlled conditions [19,21]. Workers in the rubber tyre industry, which utilises 70% of the produced carbon black, as well as those in the ink, paint manufacturing, and printing industries, may be occupationally exposed to carbon black [20].

Crystalline silica refers to minerals composed of silicon dioxide. Occupational exposure to silica is one of the most common workplace hazards. Quartz, the most thermodynamically stable polymorph under ambient conditions, is the dominant form of crystalline silica encountered in occupational settings, while cristobalite is less common [22]. Silica acts as a pollutant in various industries, including industrial production, metal mining, metallurgy, construction, tunnelling, and coal mining [23]. Exposure to silica dust in occupational environments increases the risk of numerous respiratory diseases. Workers in the ceramic industry exposed to silica have demonstrated chronic inflammatory rhinitis, nasal mucosa remodelling, and mucus thickening [24]. The respirable fraction of crystalline silica, which reaches the gas-exchange regions of the lungs, triggers chronic inflammatory responses and fibrotic changes in lung tissue, leading to silicosis and frequently progressing to lung cancer [25,26]. Since 1997, the International Agency for Research on Cancer has classified crystalline silica as a human carcinogen [22].

Nasal deposition has been studied using experimental models and computational fluid dynamics (CFD), which help interpret deposition mechanisms and identify hot spots in anatomically realistic nasal airways [1,27,28,29,30,31,32,33,34].

Building on these insights, deposition studies using nasal models provide reference data and show that deposition of micron-sized particles is strongly affected by particle inertia and inhalation flow rate [1,27,29,30,31,32,33,34,35]. However, experimental findings indicate that deposition results may vary slightly between models due to manufacturing and surface differences, which can affect inertial deposition [36].

CFD studies strengthen experimental results by providing information on intranasal airflow patterns and presenting particle deposition within defined anatomical subregions. Schroeter et al. reported strong regional diversity, with higher deposition in the nasal valve and anterior regions and lower deposition in the posterior and olfactory regions under various conditions. They used combined regional measurements with CFD to enable region-specific evaluation [32,37]. An important methodological consideration is that geometry preparation can affect deposition results. Specifically, smoothing and surface representation during model reconstruction may change inertial deposition relative to physical models, contributing to differences between CFD and experimental results [38].

Recent work has aimed to better reflect physiological conditions and comparability between studies. For example, including the external facial surface and breathing zone in the near field may change particle entry conditions and deposition patterns, especially for particles dominated by impaction, compared to entry through the nose alone [39]. Additionally, new methods have been proposed to visualise and quantify local particle deposition and to compare different geometries and scenarios [40,41]. Furthermore, Inthavong’s reviews show that CFD results depend on segmentation, verification, boundary conditions, and flow model assumptions. They recommend validation and standardised reporting for CFD in physiological or exposure-related studies [28,42,43].

Importantly for occupational exposure assessment, many nasal deposition studies have focused on therapeutic aerosols and optimised delivery (e.g., nasal sprays), while fewer studies have directly examined workplace-relevant particles [27,31,35,37,44]. For occupational evaluation, it is crucial to consider real aerosols, as workplace aerosols are often polydisperse and may contain irregular or non-spherical particles, such as fibres. CFD studies have addressed occupationally relevant particle morphologies, including fibres, and have shown relationships among deposition, particle morphology, and nasal anatomy [45,46]. Simulations of realistic workplace dusts, such as wood dust, have demonstrated deposition primarily in the anterior nasal regions, including the nasal valve and the anterior middle turbinate. They have also indicated that representing an aerosol by a single mean diameter can yield different predictions compared to using a full-size distribution [30]. Additionally, unsteady inhalation patterns can alter deposition dynamics relative to steady-state conditions [47,48,49].

Despite recent advances, experimental studies that directly compare multiple occupational aerosols in a single anatomically realistic 3D nasal model while examining steady and unsteady inhalation conditions are limited. The presented study offers a novel, occupational exposure-focused assessment of nasal deposition in an anatomically realistic three-dimensional model of the adult nasal cavity using four types of occupational dust: wheat flour, pine wood dust, carbon black, and Arizona Test Dust A3. These dusts represent polydisperse aerosols and, in some cases, non-spherical or cohesive particles relevant to workplace exposure. Normative nasal airflow variables in healthy adults, determined using CFD, can serve as a physiological reference for model-based studies [50]. Deposition was compared at controlled, steady, unidirectional inhalation rates (5, 7.5, and 20 L/min per nostril) and at an unsteady adult breathing pattern using an ASL 5000 simulator. Particle deposition was quantified using two complementary region-based indicators: segmental surface coverage (image-derived) and the segment’s share of total deposition on the lateral wall and septum, supported by additional outlet filter assessments indicating downstream passage.

Accordingly, the study addressed the following key questions: (1) How does the particle size distribution of the tested occupational aerosols govern anterior versus posterior nasal deposition under controlled inhalation conditions? (2) How do different inhalation flow conditions (steady versus unsteady) influence nasal deposition of occupational aerosol particles? (3) Which of the tested occupational aerosols pose higher risks of downstream penetration towards the lower airways?

## 2. Materials and Methods

We adapted the framework established by Rapiejko et al. [51,52] by specifically focusing on the deposition of various occupational aerosol particles and by incorporating unsteady flow conditions that were not included in the original method.

### 2.1. Material Characterisation

Four distinct aerosols relevant to occupational exposures were selected for the deposition study and generated by aerosolising powder stocks. Wheat flour type 750 (common bread flour) was chosen to represent exposures encountered by bakery workers. Pine wood sanding dust was produced using P180 fine-grained sandpaper on softwood pine specimens to simulate woodworking exposure during finishing processes. Tyre-grade carbon black, sourced from Tire Company Dębica S.A. (Dębica, Poland) was used to simulate occupational exposure in the rubber, tyre, ink, and paint industries; this material consists of highly pure elemental carbon (>95% ). Arizona Test Dust A3 (ISO 12103-1 [53] ), sourced from Powder Technology, Inc., Arden Hills, MN, USA, containing 68–76% crystalline silica, served as a standardised silica-containing dust to simulate exposure in the construction, mining, and stone-working industries. To assess the characteristics of these particles, particle size distribution (PSD) in the aerosol phase was measured using laser diffraction (Spraytec, Malvern Panalytical, Malvern, UK), with scatter-weighted steady-state averaging (transmission 80–95%). Particles were dispersed using a fluidised bed dispenser described (see Section 3.3). The reported metrics included Dv50 (median particle diameter by volume), Dv10–Dv90 range (diameters at the 10th and 90th percentiles), Span = (Dv90-Dv10)/Dv50, and volume percentage of particles smaller than 10 µm. The particle size characteristics of each occupationally relevant aerosol used in the study (Dv10, Dv50, Dv90, Span, and %V < 10 µm) are presented in Table 1 and provide the basis for interpreting differences in deposition among aerosols.

In summary, the aerosol set used in the study comprised fine mineral dust with a high proportion of <10 µm fractions (silica dust), a wider distribution of carbon agglomerates (carbon black), and typical, coarser organic and food materials (wood dust, flour). For Arizona Test Dust A3, the measured PSD of the aerosol was generally in line with the manufacturer’s specification for bulk powder (Dv50 ≈ 14 µm, Dv10 ≈ 2.6 µm, Dv90 ≈ 66 µm, ≈40%V < 10 µm), but with a slightly shortened coarse tail caused by aerosol formation.

**Figure 1:**
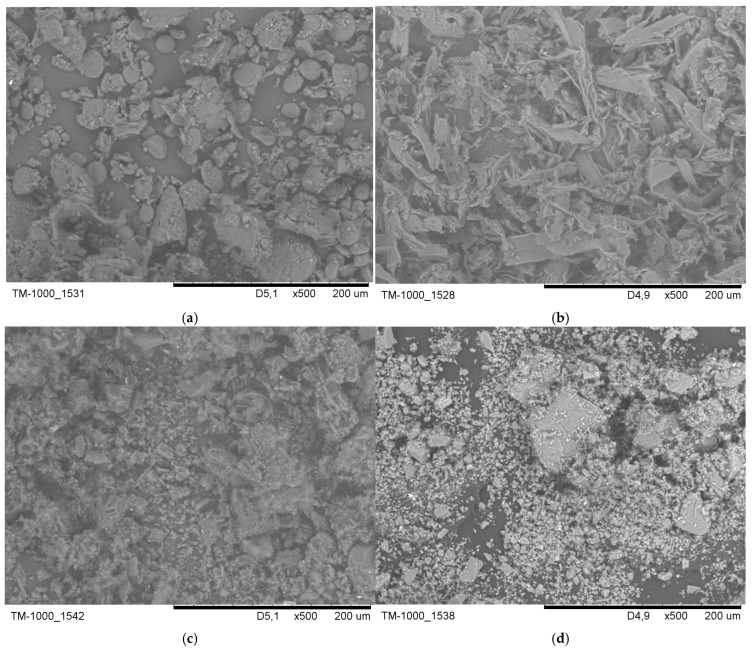
SEM images of (**a**) wheat flour, (**b**) pine wood dust, (**c**) carbon black, and (**d**) Arizona Test Dust A3 particles at 500× magnification.

To characterise the particles used in our study, scanning electron microscopy (model TM-1000, Hitachi High-Tech, Hitachinaka, Japan) was employed at magnifications ranging from 500× to 1000×. Figure 1 presents SEM (scanning electron microscope) images of the particles tested. Figure 1a shows wheat flour, which contains a polydisperse mix of smooth, rounded, or lenticular granules and irregular agglomerates, each measuring tens of micrometres. These particles have soft, non-angular contours and small satellite grains, which align with the characteristics of loosely compacted organic flour dust. Pine wood sanding dust consists mainly of elongated splinters with high aspect ratios and plate-like fragments that are tens of micrometres wide and much longer (Figure 1b). These dust particles show irregular, torn edges and rough, fibrous surfaces, typical of fractured coniferous wood cell walls. Figure 1c shows carbon black forming a dense layer of fine, strongly agglomerated particles rather than discrete grains. The dust appears as irregular, porous clusters of much smaller primary particles, in line with the fractal morphology of agglomerates. The Arizona Test Dust A3 sample is a polydisperse mixture of angular, blocky, and plate-like mineral grains, ranging in size from a few micrometres to over 100 µm (Figure 1d). The particles have sharp edges, faceted surfaces, and a compact, non-porous morphology characteristic of crushed silicate dust.

### 2.2. Anatomical Three-Dimensional Adult Nasal Cavity Model (ANCM)

The experimental methodology, previously validated by Sosnowski et al. [54], used an ANCM with geometry adapted from Bruning et al. [55]. Bruning et al. used computed tomography (CT) data from 25 asymptomatic individuals to create a reference anatomy of the nasal cavity [55]. Participants ranged in age from 17 to 57 years (mean age 37; 6 males, 19 females). Only patients with unobstructed nasal breathing, normal rhinoscopy, endoscopy, and CT scan results, without chronic nasal diseases or serious anatomical changes, were included. The authors used a semi-automated segmentation tool from ZIBAmira (v. 2015.28). Tissue was separated from the airspace by thresholding (tissue defined as Hounsfield Unit values above −400), followed by slice-by-slice segmentation using flood fill, with appropriate connected regions selected. The paranasal sinuses and ethmoid cells were excluded. Minor manual topology corrections, such as closing small holes, were applied where needed. An otorhinolaryngology specialist verified airway patency and anatomical plausibility. The surfaces then underwent volume-preserving smoothing. Smoothing-induced deviations were measured (mean distance below 0.12 mm, maximum Hausdorff distance 1.59 mm). An average (“mean”) geometry was generated using a statistical shape model. This involved 24 manually defined surface patches and PCA(principal component analysis)-based shape modelling. The resulting reference healthy-adult nasal cavity was used in our study [55].

**Figure 2:**
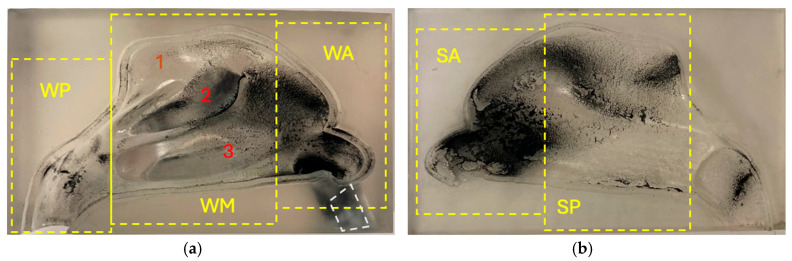
Three-dimensional prints of two parts of the anatomical adult nasal cavity model (ANCM) with carbon black deposits: (**a**) nasal lateral wall with the nasal concha (1, 2, 3) and nasopharynx; (**b**) nasal septum. Yellow rectangles indicate segments: WA—wall anterior, WM—wall middle, WP—wall posterior, SA—septum anterior, SP—septum posterior. The white dotted line indicates the location of the air inlet (the nostril, not visible in the photograph).

Based on the reference geometry, the experimental model was prepared as described below. STL (stereolithography) files for the nasal geometry were created in Blender version 4.1.10 to model the two sections and enable assembly for deposition experiments. Two identical models were printed by a 3D Form 3B+ printer (Formlabs, Boston, MA, USA) with 25 μm layer resolution, using Clear Resin V4 (Formlabs, Boston, MA, USA). The semi-transparent model was suitable for direct examination of the carbon black deposits, as shown in Figure 2. The second model was painted black to allow observation of light particles (Arizona Test Dust A3, wood dust, and flour). In line with prior studies on the deposition of nasal corticosteroids, only one part of the nasal cavity was included to effectively analyse regional deposition on the lateral nasal wall and nasal septum [56,57]. The model division followed previous designs that identified significant variability in airflow and particle deposition in these regions [29]. Before deposition testing, surfaces were coated with 1% Triton X-100 in glycerol to simulate nasal mucus and eliminate particle reflection or re-emission.

The unilateral nasal airway model was divided into 20 vertical cross-sections, each 5.25 mm apart. The first three sections (No. 1–3; 0–5.3–10.5 mm from the front) and the last two (No. 19–20: 94.8–100 mm) were not included because their airspace contours were open. Therefore, geometric measurements were taken from 15 cross-sections of the model (No. 4–18), spanning approximately 15.8–89.5 mm from the front of the model. For these measurable sections, the cross-sectional area *A* [mm^2^] and the perimeter *P* [mm] were recorded. Next, the hydraulic diameter (Dh) was calculated as Dh = 4*A*/*P*. The minimum cross-sectional area (mCSA) was determined as the minimum *A* across all analysed planes. The minimum cross-sectional area (mCSA) was 217 mm^2^, found 21.1 mm from the front; this was the lowest among measured sections. The minimum hydraulic diameter (Dh) was 3.80 mm, located 52.6 mm from the front. Across all analysed sections, the mean cross-sectional area was 295.4 ± 61.17 mm^2^, and the mean Dh was 6.88 ± 4.09 mm (mean ± SD). The Dh at the end of the WA and SA segments (3.3 cm from the front) and the WM and SP segments (7.6 cm from the front) were 5.0 mm and 17.00 mm, respectively. Finally, the volume of the unilateral model was approximately 20 mL.

We measured the pressure drop in the 3D-printed nasal model and calculated nasal resistance. The pressure drop across the model was measured using a digital manometer (TESTO, Titisee-Neustadt, Germany) and a digital flowmeter (TSI 3063, TSI Inc., Shoreview, MN, USA). The intrinsic resistance of the model has been determined to be 0.067 Pa^0.5^s/mL (the intrinsic resistance, defined as R = $\sqrt{∆P}$/*Q*, is independent of *Q*). The nasal resistance (NR), defined as NR = *∆P*/*Q*, is dependent on *Q*. The measured pressure drop across the model (inlet-outlet) and the corresponding nasal resistance values at the constant flow rates used in the experiments (5, 7.5, and 20 L/min) are presented in Table 2.

### 2.3. Experiment Process and Analysis

**Figure 3:**
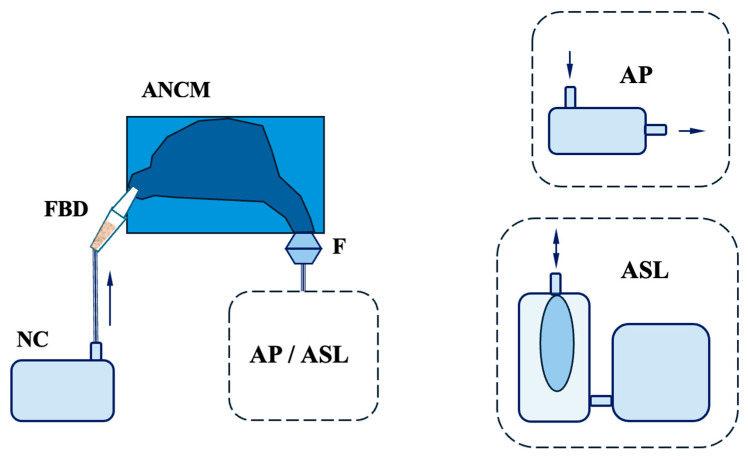
Experimental setup: NC—nebuliser compressor, FBD—fluidised bed dispenser, ANCM—adult nasal cavity model, F—outflow filter, AP—vacuum air pump (adjusted constant flow rate), ASL—breathing simulator. AP or ASL was connected optionally, depending on the type of experiment. The arrows indicate the direction of air flow in the system.

Figure 3 presents the schematic of the experimental system. Powder feedstocks (wheat flour, pine sanding wood dust, carbon black, and Arizona Test Dust A3) were loaded into a fluidised bed dispenser (FBD) and aerosolised into dry air using a medical nebuliser compressor (NC) (model Nano, Diagnostics, Diagnosis, Bialystok, Poland). FBD was constructed as a 5 mL cylindrical chamber with a conical outlet tip, allowing direct aerosol introduction into the nostril [54]. About 50 mg (±10%) of bulk powder was prepared (analytical balance—Radwag, Radom, Poland) and transferred into the dispenser. The ANCM was secured between rigid plastic plates, and marked positioning was used to ensure repeatability of measurements. The dispenser tip was then inserted into the nostril and aligned with the centre axis of the nostril to optimise delivery efficiency. The NC was operated for 5–10 s, so the full amount of powder stocks could be aerosolised and released from the FBD.

A round filter-paper disc was positioned between a protective mesh and two opposing conical covers to create a sealed, coaxially mounted cassette at the ANCM outlet. The cassette, including both filter and housing, was conditioned and weighed as a single unit and separately before and after each experimental run. The Downstream Penetration Index (DPI) was determined using the formula DPI = Δm_cassette/Δm_dispenser, where Δm_cassette denotes the mass gained by the cassette and Δm_dispenser indicates the mass lost from the powder dispenser. Photographs of the filters were examined to qualitatively evaluate the spatial distribution of the collected material.

The discharge line downstream of the ANCM was connected either to a laboratory vacuum pump (AP) or the ASL 5000 Breathing Simulator (ASL) (IngMar Medical, Pittsburgh, PA, USA) (Figure 3). In the AP configuration, the pump provided constant inhalation flow through the model, and it was adjusted prior to the experiment by a valve, with the flow rate being measured by a mass flow metre (model 4040, TSI Inc., Shoreview, MN, USA). The experiments were conducted in a unilateral configuration with inlet flow rates corresponding to different activity levels: 5, 7.5, and 20 L/min per nostril, or approximately 10, 15, and 40 L/min on both sides, assuming a perfect 50/50 split. However, the actual flow distribution may vary due to the nasal cycle and resistance asymmetry. These totals align with calm breathing (5–12 L/min), rest to light activity (15 L/min), and moderate/heavy nasal effort (40 L/min). Above about 35 L/min, the oral route commonly contributes [58].

In the ASL configuration, a spontaneous breathing simulator (ASL 5000, IngMar Medical, Pittsburgh, PA, USA) was used to simulate an unsteady, sinusoidal adult-like breathing pattern. A predefined profile called “Adult_Normal” was applied to the nasal model and used here as an unsteady nasal flow condition. The settings were URC 0.5; Rin/Rout 6 cmH_2_O/L/s; C 50 mL/cmH_2_O; sinusoidal effort; rate 15/min; Pmax 11 cmH_2_O; Increase 30%; Hold 0%; Release 10%. The following results were obtained: VT (tidal volume) = 446 mL; I:E was 1:2.0 (TI = 1.34 s, TE = 2.66 s); mean inspiratory flow = 19.8 L/min; peak inspiratory flow = 40 L/min; minute ventilation = 6.64 L/min. Therefore, AP and ASL served as alternative flow-generation modes (steady and unsteady), but the remaining experimental setup was the same. Aerosol generation lasted a split second, after which the dispenser was withdrawn from the model so that the flow necessary for aerosol formation did not affect the total flow through the nasal cavity caused by AP or ASL.

The deposition experiments at constant flow were stopped as soon as FBD was emptied. After this, both the NC and AP/ASL were switched off, and the nasal model was dismantled. Each component (lateral wall and septum) was photographed using an iPhone X (Apple, Cupertino, CA, USA). Finally, the images were analysed using proprietary Python software (v3.10.6) [54] to identify and quantify the areas of the nasal surface covered by deposits in each segment of the ANCM geometry (Figure 2). The experiments were performed in four independent repetitions (*n* = 4) for each aerosol-flow combination.

### 2.4. Statistical Analysis

Two outcome variables were evaluated. The primary outcome was segmental surface deposition (referred to as segmental deposition), calculated as the percentage of a given segment’s surface covered with occupational aerosol. The secondary outcome was the segment’s share in the total deposition on the lateral wall/segment’s share in the total deposition on the septum (referred to as lateral wall share %/septal share %), understood as the percentage of the entire lateral wall or septum surface covered with aerosol assigned to a given segment.

Segmental deposition was analysed separately for each aerosol and flow condition. For each aerosol-flow-wall combination, due to small sample sizes (*n* = 4 per group), non-parametric Wilcoxon rank-sum tests (Mann–Whitney U tests) were used to compare segmental deposition between anterior and posterior segments (lateral wall: WA vs. WP; septum: SA vs. SP), as well as between the middle and posterior segments on the lateral wall (WM vs. WP). A one-tailed alternative hypothesis (more anterior segments > posterior) was tested based on the expectation that deposition would decrease toward the rear, with two-tailed *p*-values also calculated for completeness. The effect of flow rate on deposition in individual segments was assessed using one-way analysis of variance (ANOVA) with airflow rate as a factor, separately for each aerosol and wall. Homogeneity of variance was assessed using Levene’s test. Effect sizes (η^2^) were calculated, and according to Cohen, values ≥ 0.14 are considered large. In cases of a significant main effect of flow, pairwise differences between flows were examined using Tukey’s Honestly Significant Difference (HSD) post hoc test. ANOVA and post hoc analyses were performed for WA, WP, SA and SP, which were selected a priori to assess the anterior–posterior gradient, while WM was presented descriptively (median, IQR). Descriptive results were reported as medians and interquartile ranges [IQR (Q1–Q3)] in Section 3.2.

The second variable, lateral wall share%/septal share%, mathematically related to the first variable and the segments’ surface area, was treated as a complementary outcome to describe how aerosol type and flow redistribute total deposition between segments. A two-way ANOVA was conducted with aerosol type and flow as fixed factors. For the lateral wall, a multivariate ANOVA (MANOVA) was performed across all segments, and separate one-way ANOVAs were conducted for each segment. Additionally, within each aerosol, the Kruskal–Wallis test was used to assess the effect of flow, with Dunn’s test (Bonferroni correction) for post hoc comparisons, and extreme flows (20 vs. 5 L/min) were compared using Welch’s *t*-test. Assumptions of homogeneity of variance were assessed using Levene’s and Bartlett’s tests. Detailed median and IQR values for lateral wall share% and septal share% are provided in the Supplementary Materials. Statistical significance was set at α = 0.05. The analyses were performed in the R environment.

## 3. Results

### 3.1. General Characteristics of Segmental Deposition

Segmental deposition on the lateral wall and septum at each flow rate (5, 7.5, 20 L/min per nostril), as well as during unsteady breathing (ASL), varied significantly among segments for each aerosol tested. Across all flow rates and particle types, anterior deposition was the predominant mode. For all aerosols analysed, segmental deposition was significantly greater in the anterior segment (WA, SA) than in the posterior segment (WP, SP) on both the lateral wall and the nasal septum. On the lateral wall, deposition in the middle segment (WM) also exceeded that in the posterior one, regardless of flow rate (Wilcoxon rank-sum test, *n*1 = *n*2 = 4, W = 16, one-sided *p* < 0.02; two-sided *p* = 0.029). Median segmental deposition in WA ranged from approximately 38% to 70%, in WM from 15 to 19%, and in WP from 4 to 9%. On the septum, median segmental deposition in SA ranged from approximately 42% to 67%, and in SP from 12% to 18%. Aerosols with a larger sub-10 µm fraction, such as Arizona Test Dust A3 and carbon black, produced relatively higher WM and WP deposition compared to wheat flour type 750 and pine wood sanding dust, though WA and SA remained consistent main sites.

### 3.2. Air Flow Dependence

The increase in flow primarily led to increased segmental deposition in the anterior segments of both the lateral wall and the septum. Deposition in WA increased significantly with increasing flow rate for all aerosols (ANOVA, *p* < 0.001) with large effect values (η^2^ = 0.6–0.96). For the septum, a similar relationship was also observed for SA (ANOVA: Arizona dust, wheat flour, wood dust, *p* < 0.001; carbon black, *p* = 0.009). Tukey’s post hoc analysis showed that the values for 20 L/min were significantly higher than for 5 and 7.5 L/min for all aerosols (Tukey HSD, *p* < 0.01), and the flow rate of 7.5 L/min gave higher values than at 5 L/min (*p* ≤ 0.04).

In the posterior segments, the effect of flow was weaker and different. On the lateral wall, a small but significant effect of flow was found for WP for all aerosols (ANOVA, Arizona dust *p* < 0.001; wheat flour *p* = 0.017; wood dust *p* = 0.009; carbon black *p* = 0.005). Median deposition in WP decreased with increasing flow, indicating a shift in the zone of highest deposition towards the anterior part of the nasal cavity. Deposition at SP remained essentially stable; no significant differences were found between flows in SP for Arizona Test Dust A3, pine wood dust, and carbon black (ANOVA, *p* = 0.14; *p* = 0.55; *p* = 0.19, respectively). However, for wheat flour, a small but significant increase in deposition was observed at higher flows (*p* = 0.001), mainly between 5 and 20 L/min (Tukey HSD, *p* < 0.01). In post hoc analyses on the lateral wall, the differences mainly concerned comparisons of 5 vs. 20 L/min (Tukey HSD, *p* < 0.02). The other flow pairs often did not reach statistical significance. WM showed intermediate values and changes with flow between WA and WP.

Unsteady flow usually gave intermediate values, significantly higher than at 5 L/min and often higher than at 7.5 L/min (*p* < 0.001–0.02), and not significantly different from 20 L/min (*p* > 0.20). Qualitatively, they were mostly between values for 7.5 L/min and 20 L/min. WA and SA values were closer to 20 L/min, showing the effect of peak inspiratory flow. WP values matched the 7.5 L/min setting due to a longer inspiratory phase.

The observed anterior shift (WA/SA↑; WP↓) with the increase in flow rate aligns with increased inertial impaction in the valve and turbinate region. When we assessed the impaction parameter under our measurement conditions using the median diameter of the polydisperse aerosol (IP = Dv_50_^2^·Q), IP ranged from 10^4^ to 10^6^ (µm^2^cm^3^/s), indicating the dominant influence of the inertial mechanism on particle deposition. This trend was consistent for all aerosol types, particularly for larger particles such as wheat flour and pine wood dust, and to a qualitatively weaker extent for Arizona Test Dust A3. SP changes were minor, meaning only wheat flour showed a clear increase, while other aerosols showed at most a slight minimum of around 7.5 L/min, with no statistically significant differences.

**Figure 4:**
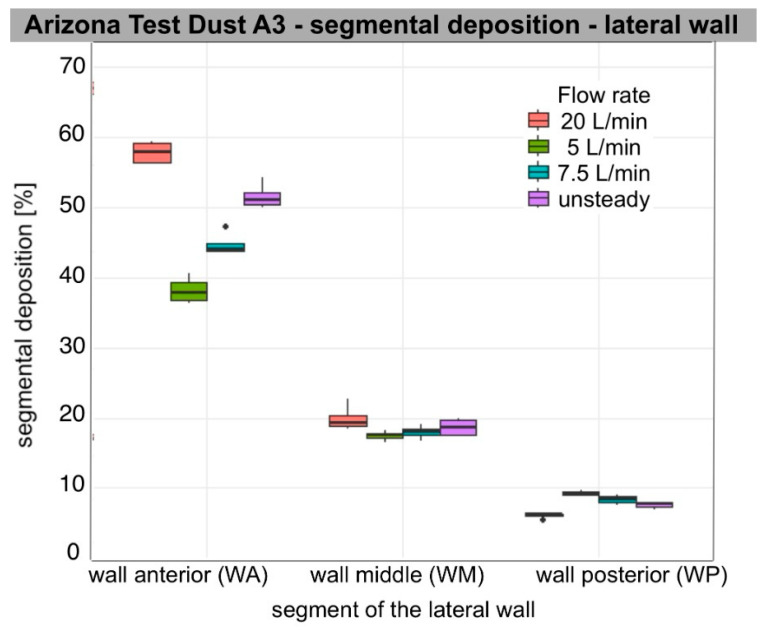
Segmental deposition for four flow rates (5, 7.5, 20 L/min, unsteady) of Arizona Test Dust A3 on the lateral wall. Box plots are based on four independent repetitions per aerosol-flow condition (*n* = 4). Black dots represent outliers, i.e. individual observations outside the whiskers.

**Figure 5:**
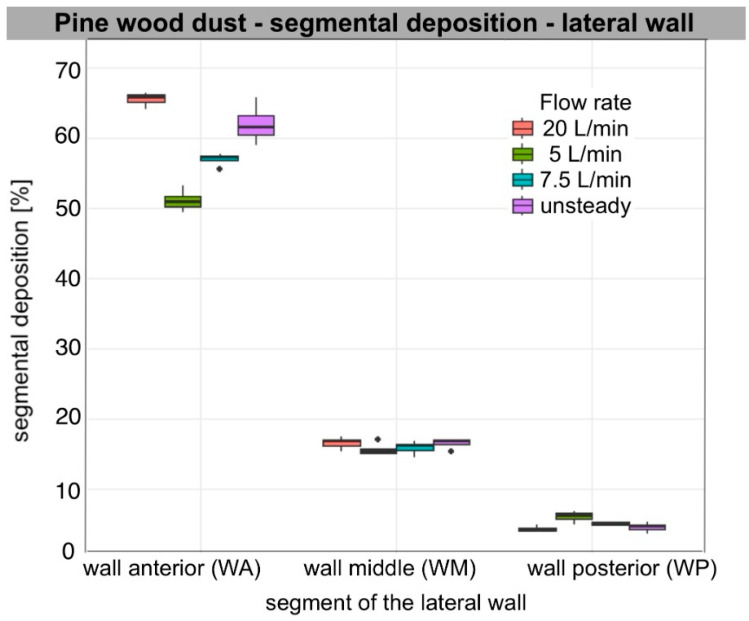
Segmental deposition for four flow rates (5, 7.5, 20 L/min, unsteady) of pine wood dust on the lateral wall. Box plots are based on four independent repetitions per aerosol-flow condition (*n* = 4). Black dots represent outliers, i.e. individual observations outside the whiskers.

**Figure 6:**
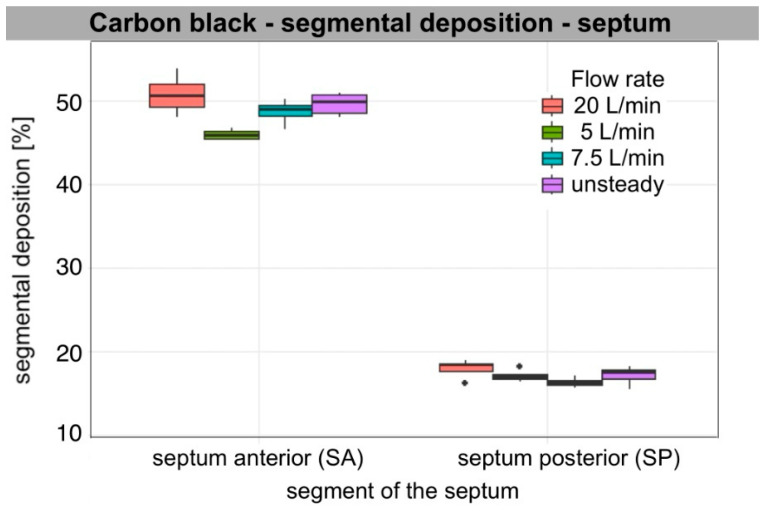
Segmental deposition for four flow rates (5, 7.5, 20 L/min, unsteady) of carbon black on the septum. Box plots are based on four independent repetitions per aerosol-flow condition (*n* = 4). Black dots represent outliers, i.e. individual observations outside the whiskers.

**Figure 7:**
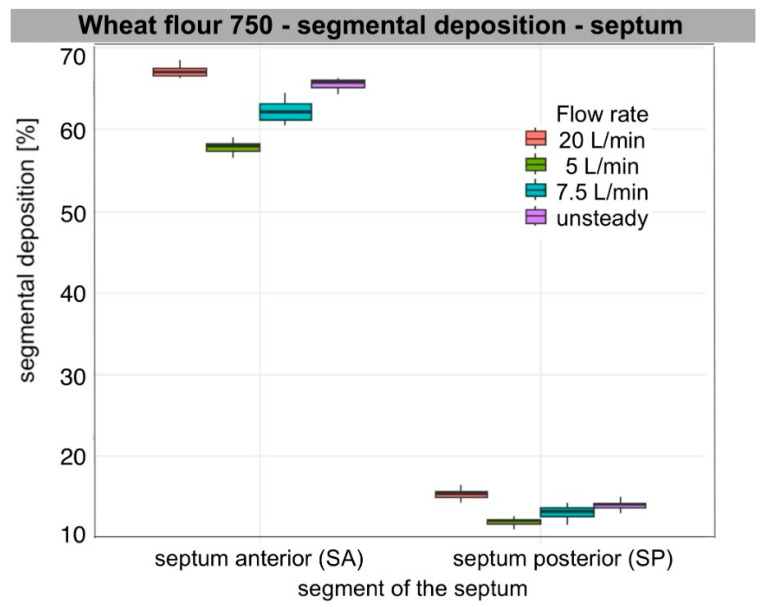
Segmental deposition for four flow rates (5, 7.5, 20 L/min, unsteady) of wheat flour type 750 on the septum. Box plots are based on four independent repetitions per aerosol-flow condition (*n* = 4).

Table 3 presents descriptive results (median, IQR [Q1–Q3]) of segmental deposition of occupationally relevant aerosols, expressed as the percentage of segment surface area on the lateral wall and septum. The ANOVA results in Table 4 illustrate the relationship between deposition and flow rate within individual segments. The distribution of the data is presented in box plots (Figure 4, Figure 5, Figure 6 and Figure 7).

### 3.3. Characteristics of Lateral Share %/Septal Share % Deposition

Analysis of the second variable confirmed the pattern obtained for segmental deposition. The segment’s share in the total deposition on the lateral wall depended significantly on aerosol type and flow, without significant aerosol-flow interaction (ANOVA: aerosol F(3,48) = 98.2; *p* < 0.001; flow F(3,48) = 30.2; *p* < 0.001; interaction *p* = 0.21). MANOVA and one-way ANOVA for each segment confirmed the significant impact of both factors on the distribution of total share % between segments. In non-parametric analyses, the effect of flow was significant for Arizona Test Dust A3, wood dust, and wheat flour (Kruskal–Wallis *p* ≤ 0.04), with only a trend for carbon black (*p* = 0.072). For the WP, the mean contribution was smaller at 20 L/min than at 5 L/min (0.0085 vs. 0.0117 of total area; Welch’s *t*-test, t = −4.03; *p* = 0.00038), which indicates a relative decrease in WP contribution at higher flows.

Septal share % was mainly determined by the type of aerosol and, to a lesser extent, by the flow, also without significant interaction (ANOVA: aerosol F(3,48) = 52.5; *p* < 0.001; flow F(3,48) = 7.83; *p* = 0.00024; interaction *p* = 0.10). A significant effect of flow was found only in the non-parametric analysis for wheat flour (Kruskal–Wallis χ^2^(3) = 11.41; *p* = 0.0097); for the other aerosols, the differences were insignificant. Comparison of 20 vs. 5 L/min for septal share % showed only a trend towards a higher mean value at the highest flow rate (0.123 vs. 0.112; Welch’s *t*-test: t = 1.93; *p* = 0.064). Overall, these results confirm that most of the deposited aerosol mass is concentrated in the anterior and middle parts of the nasal cavity, and at higher flows, the relative contribution of the posterior segments decreases further.

For carbon black, Arizona Test Dust A3, and pine wood dust at 5 L/min, qualitatively, WM was the contributor, given its largest area. WA contributed substantially less, and WP contributed minimally (1%). For pine wood dust at 7.5 and 20 L/min and under an unsteady breathing pattern, and for wheat flour across flows, WA accounted for the largest lateral share (%), WM less, and WP remained negligible. On the septum, for carbon black and Arizona Test Dust A3, the SP segment’s share of the total septum deposition was bigger than SA’s, due to SP’s larger surface area. However, for larger particles, such as wheat flour and pine wood dust, SA was dominant. As flow increased, WA and WM lateral share % increased, while WP’s share decreased. For non-steady flow, values for each segment ranged from 7.5 L/min to 20 L/min. Detailed median [IQR] values for segment share of total lateral wall and septal deposition for each aerosol and flow condition are presented in the Supplementary Table S1.

### 3.4. Characteristic Features of the Results of Individual Aerosol Deposition

Segmental patterns were qualitatively consistent with the measured particle size distribution (PSD) metrics summarised in Table 1. For all materials, smaller Dv50 values corresponded to increased WM and WP deposition at lower flows (5–7.5 L/min), while larger Dv50 values led to increased WA and SA and minimal WP across all flows. The proportion of sub-10 µm particles further distinguished segmental deposition, as a higher percentage of particles below 10 µm was associated with increased WM and WP at lower and medium flow rates. The difference between 5 and 7.5 L/min was small, but at 20 L/min, anterior impaction dominated, reducing this effect. Additionally, a lower Dv10 resulted in higher WM and WP and less sensitivity to changes from 5 to 7.5 L/min. Conversely, higher Dv90 values, indicating a greater number of very large particles, resulted in increased WA and SA, especially at 20 L/min, and increased variability in the anterior segments due to sporadic large-particle impacts.

Among the aerosols, Arizona Test Dust A3 (Dv50 = 15.4 µm; Dv10 = 3.2 µm; 36.4% < 10 µm) exhibited high WM and WP at flow rates of 5 to 7.5 L/min. There was a shift toward higher WA and SA, accompanied by a marked decrease in WP at a flow rate of 20 L/min. Carbon black (Dv50 = 23.4 µm; Dv10 = 5.4 µm; 22.9% < 10 µm) showed a similar trend, but WM/WP was lower than that of Arizona Test Dust A3. As the flow rate increased, WA and SA increased. WP decreased, while WM stayed relatively constant. Wheat flour (Dv50 = 40.6 µm; Dv10 = 16.8 µm; 3.1% < 10 µm) and pine wood dust (Dv50 = 43.3 µm; Dv10 = 15.5 µm; 4.3% < 10 µm) both exhibited dominant WA and SA, with minimal WP at all flow rates. For both, a higher Dv90 further increased WA and SA at 20 L/min. Pine wood dust, due to its flaky, fibrous morphology, produced clustered or striped deposition patterns. This resulted in lower areal coverage compared to more isotropic aerosols with similar Dv50 values. Wheat flour, a hygroscopic material, created a more diffuse deposition footprint and achieved higher areal coverage at comparable Dv50 values. Both carbon black and Arizona Test Dust A3 exhibited uniform and granular deposition patterns, consistent with near-isotropic impaction behaviour.

### 3.5. Descriptive Rank Order of Deposition (By Medians)

Cross-material differences are summarised in a qualitative rank order based on medians and interquartile ranges, providing a framework for interpretation and comparison. In the anterior segments (WA/SA) at constant flows (5 to 20 L/min), the highest values occurred in aerosols with larger size fractions and fewer particles below 10 µm: wheat flour ≥ pine wood dust > carbon black ≥ Arizona Test Dust A3. In the nasopharynx (WP) at 5 to 7.5 L/min, the trend reversed: Arizona Test Dust A3 > carbon black >> wheat flour ≈ pine wood dust. At 20 L/min, WP remained low for all, with the order: Arizona Test Dust A3 ≥ carbon black > wheat flour ≈ pine wood dust. In the middle segment (WM), Arizona Test Dust A3 ≳ carbon black > wheat flour ≈ pine wood dust persisted, though differences were smaller than in WP. The SP reflected the WM order. With the ASL (unsteady) profile, levels were intermediate: WA and SA resembled 20 L/min, and WP and SP paralleled 7.5 L/min. The ranking order among materials remained stable.

### 3.6. DPI (Downstream Penetration Index) and Filter Appearance

**Figure 8:**
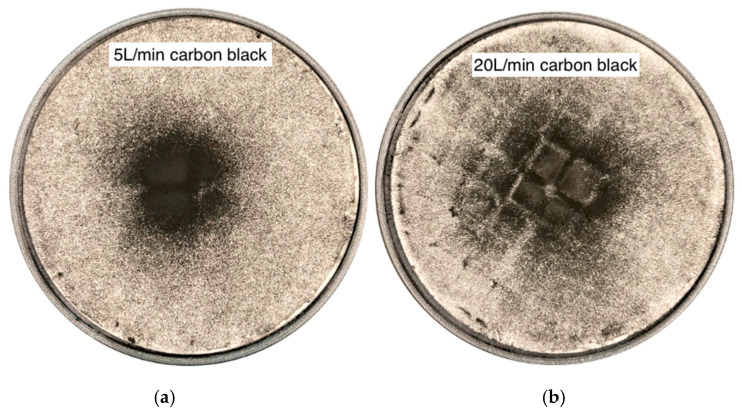
Representative filter photographs for carbon black at 5 L/min (**a**) and 20 L/min (**b**).

The Downstream Penetration Index (DPI), measuring the ratio of filter mass to dispenser loss, was calculated, and the resulting values are reported in the Supplementary Table S2. DPI decreased as flow rate increased for all aerosols (5, 7.5, 20 L/min). At 5 L/min, Arizona Test Dust A3 had the highest DPI, followed by carbon black; pine wood dust and wheat flour had the lowest. This ranking persisted as flow rose, with DPI values decreasing overall. Under unsteady breathing (ASL), DPI values were intermediate between those at 7.5 and 20 L/min. Differences among materials were reflected in PSD metrics (Table 1): Arizona Test Dust A3 (Dv50 = 15.40 μm) consistently demonstrated higher downstream penetration than pine wood dust, wheat flour, and carbon black. The DPI hierarchy (Arizona Test Dust A3 > carbon black >> wheat flour ≈ pine wood dust) was consistent at all flows. Comparative images in Figure 8 show that, following carbon black deposition, the downstream filter at 5 L/min had a small, central, dense spot (a focused jet with minimal dispersion). At 20 L/min, the spot became larger and more diffuse, with a lower optical density per area, and the filter grid was visible, indicating a broader and more turbulent plume. As the flow increased, the spot diameter expanded, and local darkness decreased, suggesting that greater anterior impaction at higher flow reduces DPI and that spot size is not directly correlated with the mass captured.

## 4. Discussion

This study extends previous research on particle deposition in the nasal cavity by mapping segmental and area-weighted patterns for four occupationally and environmentally relevant aerosols (wheat flour, pine wood dust, carbon black, and Arizona Test Dust A3), which are commonly encountered in different occupational settings and have significant health risks. It compares constant flows (5, 7.5 and 20 L/min) and unsteady breathing pattern and builds on our previous research on pollen, taking into account polydisperse, non-spherical, cohesive powders, as well as direct comparisons between steady (model) and unsteady (realistic) inhalation [51,52]. For these aerosols, deposition occurs mainly in the anterior part of the nasal cavity, especially with greater inspiratory demand, and is influenced by particle size.

### 4.1. Mechanistic Explanation of Nasal Deposition

The mechanisms of aerosol deposition in the nasal cavity are well-known, and they are similar to those in other separation systems, such as filters. Here, we briefly summarise the mechanisms most relevant to the particle size range and breathing conditions investigated in this study. In brief, large airborne particles (20–50 µm) inhaled at high velocity have high inertia; therefore, their primary deposition mechanism is inertial impaction with obstacles when airflow changes direction abruptly. In the nasal cavity, impaction occurs in the anterior parts of the lateral wall and the septum. This happens when airflow aligns before entering deeper parts of the nose. This mechanism is predominant for the particles studied in this work. It is also obvious that particles already removed from the air in the anterior parts of the nose will not be deposited in the deeper structures. Common parameters used to assess inertial deposition include the Stokes number and the impaction parameter.

Gravitational sedimentation, which is also important for large particles, requires a longer residence time in the system, which cannot be achieved in the nose. Therefore, this mechanism is expected to be relatively minor under the present conditions. Nasal deposition is not affected by the electrostatic mechanism, since the inner surface of the nose is covered by mucus, which is a conductive liquid and cannot accumulate an electric charge. Brownian diffusion is important only for ultrafine particles, which were not present in the aerosols studied in this work. A mechanism that should be considered in the aerosols studied is direct interception. This mechanism applies to nonspherical particles, such as needle-like or fibrous particles, which can penetrate deeper due to reorientation as they pass obstacles. However, they can contact the nasal surface in deeper segments because of their dimensions.

The presented mechanisms enable us to rationally discuss the deposition distribution obtained for different types of polydisperse environmental particles studied here, inhaled under various conditions; however, it is not possible to achieve the same level of numerical precision as in CFD simulations. Nevertheless, the experimental data can be used in conjunction with the modelling results to better understand the physics of nasal deposition and its potential health effects.

### 4.2. Steady Airflow

The observed deposition patterns (dominance of WA/SA, moderate WM, minimal WP/SP) are typical for aerosols with sizes ranging from several to several dozen micrometres, where the primary mechanism of deposition is inertial impaction, which follows the dimensionless Stokes number.

(1) $$Stk=\frac{u\rho d^{2}}{18\mu L}$$

where *u* [m/s] means the velocity of the particle (assumed equal to the velocity of inhaled air), *ρ* [kg/m^3^]—particle density, *d* [m]—particle effective diameter, *μ*—air viscosity [kg/(m s)], and *L*—the air channel’s characteristic size [m]. Alternatively, inertial deposition can be characterised by inertial parameter IP = *d*^2^*Q* [1]. Higher Stk values indicate a stronger influence of particle inertia, causing particles to deposit. As the flow increases, *u* and Stk values rise, causing particles to deviate from streamlines at curvatures and sudden turns, particularly in the nasal vestibule and the front part of the septum, resulting in the observed anterior dominance (WA/SA) and enhancement at higher flow velocities. In contrast, when Stk is smaller (finer fractions, lower flow), the particles follow the streamlines for a longer time and are captured in the WM and sometimes in the WP. Hence, relatively greater deposition was found in the middle and posterior sections for aerosols with a larger fraction below 10 µm at 5–7.5 L/min. With the increase in flow, deposition was redistributed towards the front: a strong increase in segmental deposition was observed in the front segments (SA/WA), while in the posterior segments of the lateral wall (WP), there was a small but significant decrease, and deposition in the SP remained largely stable. These relationships between flow and anatomy reflect the dominance of the anterior region, which we reported earlier in relation to pollen and which has now been reproduced for occupational aerosols [51,52]. CFD studies report that the largest pressure drop occurs in the anterior narrowing (nasal valve or isthmus nasi region), while the posterior turbinate region contributes less to the overall pressure drop [50,55]. The findings are consistent with in silico and in vitro studies demonstrating that nasal deposition increases with both particle size and flow, and that hotspots shift towards the front (valve, anterior/middle turbinate) as flow increases [1,29,30,31,34]. The CFD analysis of 2–60 µm particle deposition in the nasal cavities of newborns, infants, and adults, performed by Corda et al., revealed that the nasal valve and vestibule play a major role in particle deposition, especially for particles larger than 10 µm, and as particle size increases, deposition shifts to the front [27].

### 4.3. Particle Size

Numerous in silico and in vitro studies have shown that nasal deposition is influenced not only by median particle size but by the full particle size distribution. Aerodynamic behaviour of large aerosols with high Dv50 and Dv90 values and negligible fine fraction is dominated by inertia, so these particles mostly deposit in the vestibule, nasal valve, and anterior septum/turbinates and have very limited penetration into the nasopharynx. In contrast, aerosols with smaller diameters and a significant sub-10 µm fraction can partially follow the airflow at low or moderate airflow rates. This allows them to reach the middle and posterior segments and also penetrate beyond the nasal cavity. Deeper deposition aligns with the fact that, for smaller sizes, mechanisms other than inertial impaction—interception for non-spherical particles and gravitation sedimentation (depending on residence time and local velocities)—and Brownian diffusion become relevant only for ultrafine particles [1,27,30,34,59]. Accordingly, the PSD characteristics reported in Table 1 (Dv10, Dv50, Dv90, Span and %V < 10 μm) were used in the study to interpret the observed differences in deposition and downstream penetration among tested aerosols.

Tian et al., using the CFD technique, found that pine and oak dust (3–55 μm) deposited mainly at the nasal valve and the anterior part of the middle turbinate at 10 L/min [30]. The authors noted that wood dust deposited in these areas was removed more slowly than in other areas. This damages the surrounding soft tissue layer due to the deposition of particles over time. A small fraction of particles was also deposited in the posterior part of the nasal cavity due to the flow changing direction to vertical downward. In addition, pine dust showed higher deposition efficiency than oak dust, showing that materials richer in coarse particles exhibit higher deposition efficiency in the anterior part, while dusts with a higher fine fraction penetrate further into the posterior parts [30].

Shen et al. studied deposition of lactose particles (10–109 μm) at 15–55 L/min and showed that the anterior part of the nasal cavity exhibited the greatest deposit thickness at all flow rates [34]. Smaller particles were distributed more evenly and extended posteriorly, while thicker particles accumulated at the front. A review of CFD studies by Guo et al. reported that nasal deposition is proportional to particle size and airflow, with coarser fractions depositing mainly anteriorly, while finer fractions show a relatively higher proportion in the middle and posterior regions at low to moderate flows [1].

This framework explains observed deposition patterns for tested aerosols, including wheat flour and pine wood dust (with high Dv50/Dv90 values and negligible particles < 10 µm), which consistently produce high WA/SA values and minimal WP values, which agrees with the dominant influence of coarse particle inertia. In contrast, Arizona Test Dust A3 and carbon black (larger fine fraction) showed higher WM/WP values at 5–7.5 L/min, reflecting the ability of their fine fraction to penetrate beyond the nasal valve and deposit on the middle and posterior walls.

### 4.4. Unsteady Airflow

The use of a breathing simulator enables a more accurate representation of physiological breathing patterns and their impact on particle deposition. Physiological breathing involves unsteady airflow; each cycle consists of acceleration, a short peak inspiratory flow (PIF), and an extended phase of deceleration, allowing inhaled particles to encounter a variable velocity field. Studies based on the CFD technique or experimental casts show that steady-state simulations can estimate overall nasal deposition for specific particle size ranges but may misrepresent regional deposition and penetration, particularly for micron-sized particles. Large, inertia-dominated particles are predominantly deposited during the short, high-flow phase, particularly in the anterior nasal cavity regions. On the other hand, smaller, low-inertia particles (usually less than 10 μm) are affected by residence time and recirculation zones in the main nasal passage. Longer periods of moderate flow during the respiratory cycle allow these particles to reach the middle and rear parts of the nasal cavity [47,48,49,60,61,62].

Haußermann et al. found that for fine particles (1.7–3 µm), total nasal deposition was higher during steady flow than during an unsteady breathing pattern, while the effectiveness of deposition at 10 L/min was similar to cyclic flow [60]. Bahmanzadeh et al. reported, using CFD, that for very small particles (less than 10 µm), steady-flow conditions tended to overestimate nasal and upper-airway deposition compared with cyclic breathing at equivalent average flow rates. For particles in the 15- to 20-µm range, cyclic breathing produced slightly higher deposition than steady flow. For larger particles (over 25–30 µm), steady-state simulations likely overestimated deposition due to a momentum-driven rebound or particle losses during exhalation [48]. In turn, Se et al., using the CFD, found that unsteady inhalation slightly reduced the total deposition of 20 µm particles compared to an equivalent steady flow and increased the share of deposition in the middle region. For 5 µm particles, overall deposition remained nearly unchanged; although regional deposition patterns were similar, differences were noted in timing and escape fraction [47].

Naseri et al. emphasised that the exhalation phase is important, as particles may be exhaled or deposited during this period. In their numerical studies, regional deposition patterns showed that deposition fractions in the vestibule, main airway, and nasopharynx were higher during cyclic breathing than during steady flow, whereas the opposite was true for the oropharynx, larynx, and trachea. For finer particles (less than 15 μm), cyclic flow resulted in slightly higher nasal cavity deposition than steady flow. For larger particles (15–30 μm), steady flow showed slightly higher deposition fractions, while at the same time, deposition extended toward the central and posterior regions, resulting in a more distributed, phase-dependent pattern [61].

Yu et al., in their numerical analysis of the migration and deposition characteristics of microdust in the upper airways, showed that the nasal escape rate during exhalation was around 90% for 1 μm particles but less than 2% for 15 μm particles and practically 0% for particles above 20 μm [35]. It suggests that coarse aerosols accumulate in the anterior nose and cannot be effectively removed from the nose by exhalation, while fine-rich aerosols show lower nasal deposition and may partly clear during exhalation.

In our study, all flow conditions were applied to a unilateral nasal model. For steady inhalation, flow rates of 5, 7.5, and 20 L/min per nostril corresponded to approximate bilateral flows of 10, 15, and 40 L/min, respectively. In contrast, the ASL adult profile produced a mean inspiratory flow of 19.8 L/min and a peak inspiratory flow (PIF) of 40 L/min per nostril, equivalent to 40 and 80 L/min bilaterally. This unsteady condition does not represent exactly resting bilateral nasal breathing, but instead, it reflects physiologically plausible scenarios, such as unilateral breathing during a nasal cycle phase when one nostril is predominantly patent and receives most of the ventilation, or light to moderate exertion with bilateral peak flows reaching 80 L/min. Both scenarios are relevant in occupational settings, where workers perform light physical tasks or may suffer from dust-exposure-induced rhinitis and transiently experience similar flow patterns.

Consistent with unsteady flow simulations, segmental deposition under ASL conditions was intermediate between steady-state results at 7.5 and 20 L/min. The anterior segments (WA/SA) showed deposition values approaching those observed at 20 L/min, suggesting that peak inspiratory flow mainly causes impaction. In contrast, deposition in the nasopharynx (WP) remained closer to that observed at 7.5 L/min, as most of the respiratory cycle occurred at moderate flow velocities. The relative order of aerosol types remained unchanged.

### 4.5. Aerosol Particle Morphology

SEM imaging confirmed morphological differences among particles examined. Wheat flour formed rounded granules and loose agglomerates. Pine wood dust consisted of elongated splinters. Arizona Test Dust A3 appeared as angular mineral grains, while carbon black was observed as highly agglomerated clusters. Morphological differences affect the same fundamental deposition mechanisms [1,59]. Elongated softwood splinters enhance interception and orientation-dependent inertial impaction [30,63]. Angular mineral grains promote sharp-surface inertial impaction in the anterior nose and pharynx [64,65]. Fractal black carbon agglomerates exhibit aerodynamic behaviour characteristic of coarser particles than their primary size indicates; however, they primarily deposit through impaction and, for ultrafine fragments, diffusion [66].

In addition to particle size distribution, particle density (*ρ*) impacts deposition, affecting the Stokes number (Equation (1)), but its influence, together with velocity (*u*), is linear and weaker than the quadratic relationship with particle size (*d*^2^). Although moisture content can increase the effective particle density in vivo, its influence is less significant than geometric size. The characteristic dimension of the airways (*L*) amplifies the effect of density in narrow areas (the Stokes number increases for smaller *L* values). This explains the high deposition in the nasal valve and turbinates, sites of geometric constriction, even for particles of moderate density.

A recent review of the literature on the application of the CFD technique by Guo et al. showed that the total nasal deposition increases with particle size and density: gradually for particles with a diameter of 2.5 µm and rapidly for particles with a diameter of 5 µm [1]. Furthermore, Yu et al. found that, at a fixed geometric size, a higher density of 15 µm coal and rock dust significantly reduced nasal escape during exhalation. This indicates that medium-sized, high-density particles are mainly retained in the nasal cavity and pharynx (if they penetrate there). Large particles (≥20 µm) were retained in the upper respiratory tract regardless of their density [35]. A similar trend was observed for wood dust. Simulations by Tian et al. showed that heavy oak dust settled in the nasal cavity to a greater extent than light oak dust of the same size. This may result from lower-density dust having a lower Stokes number and therefore following the airflow better [30]. Sun et al. showed that among 5 µm occupational dusts, nasally inhaled very dense, nearly spherical copper dust showed the highest total deposition in the larynx and bronchi, while aerosols with lower densities, such as rock, coal, and wood dust, showed higher nasal deposition and lower deposition in the lower respiratory tract [67]. The observations of softwood dust are consistent with established fibre mechanics for elongated particles in the nasal cavity.

Fibres, which are dominated by inertial forces, form a persistent deposit hotspot in the anterior part of the vestibule and the anterior part of the septum. Fibre orientation and rotation often result in reduced inhalability and non-uniform, striped deposition patterns compared to more isotropic powders with similar Dv50 values [46,68]. Studies comparing carbon fibres with titanium dioxide and glass fibres in nasal replicas confirmed this distinction between fibres and spheres. High-inertia carbon fibres are primarily deposited in the anterior region of the nasal cavity, whereas low-inertia fibres penetrate deeper into the lower respiratory tract [63]. Additionally, in vitro and CFD studies have shown that increasing fibre length or aspect ratio reduces penetration beyond the valve and turbinate area, and higher flow rates enhance anterior impaction [46,68]. Recent CFD studies on *Platanus orientalis* fibres further showed that, at a constant diameter, longer fibres had a larger aerodynamic diameter and were less inhalable. At a constant aerodynamic diameter, fibres with a high aspect ratio entered the nose more easily than spheres. Still, they settled mainly in the vestibule and anterior part of the nasal septum [68].

These mechanisms explain the high WA/SA and moderate WM/WP deposition patterns observed for pine wood dust in our study, despite its median particle size resembling that of wheat flour. Spherical or nearly spherical hygroscopic powders, like wheat flour, have no orientation restrictions and tend to spread more evenly in the anterior part of the nasal cavity. For example, while pine wood dust formed concentrated, striped deposits in the anterior part of the nasal cavity, flour exhibited a diffuse, uniform pattern. Carbon black and Arizona Test Dust A3 exhibited granular, nearly isotropic patterns, consistent with nearly isotropic impaction. This behaviour is consistent with the literature, which shows that cohesion, aggregation, hygroscopic growth, and particle shape influence particle transport and deposition. These results also suggest that, in addition to particle morphology, spherical optical parameters should be considered [46,57]. In vivo, the hygroscopic growth of flour particles in humid air within the nasal cavity may further increase their effective aerodynamic size and lead to greater anterior nasal deposition.

Many physical phenomena influence the deposition of aerosol particles. For aerosols larger than 20 μm, the main mechanism of deposition is inertial impaction. Gravitational settling and electrostatic forces are negligible due to the short residence time and moist (i.e., conducting for the electrical charge) surface of the mucosal surfaces. However, direct interception, meaning contact between a particle and the airway wall, remains significant for aggregates larger than 100 μm in diameter. As a result, light, non-spherical clusters can deposit in the middle and posterior regions despite low Stokes numbers [59].

### 4.6. DPI

The Downstream Penetration Index (DPI) serves as a quantitative measure of nasal filtration efficiency. In our data, the DPI decreased systematically with increasing flow for all aerosols, which is consistent with inertial impaction as the dominant mechanism for the particles studied. This mechanism promotes particle deposition in the anterior regions of the nasal cavity, and fewer particles reach the filter. These results are consistent with experimental and CFD studies showing stronger anterior filtration and reduced particle penetration at higher flow rates [30,31,34,64]. Studies by Cheng et al. on coal dust found that the escape rate decreased with increasing particle size and work intensity [64]. Furthermore, Peng et al. showed that higher breathing intensity reduces nasal deposition of coal dust smaller than 10 µm while increasing deeper penetration [65].

The material-specific DPI behaviour reflects both the particle size distribution (PSD) (Table 1) and the effective aerodynamic properties. Wheat flour and softwood dust, with high Dv50 values and small fractions below 10 µm, are effectively filtered in the anterior nasal cavity and show the lowest DPI values at all flow rates. Coarse wood dust and fibrous particles are deposited mainly in the vestibule and anterior part of the nasal septum, with limited penetration into the deeper airways [30,46,69]. In contrast, Arizona Test Dust A3, which contains the largest fraction of particles smaller than 10 µm, exhibits the highest DPI at every flow rate. Carbon black, characterised by a wider size distribution and a moderate proportion of fine particles, yields intermediate DPI values. The intermediate DPI of carbon black aligns with its porous, fractal agglomerates, which exhibit aerodynamically coarser behaviour than their primary particle size suggests, while still contributing a substantial sub-10 µm volume fraction.

Visual analysis of deposits on filter paper supplemented the DPI measurements. At low flow rates (5 L/min), carbon black formed a compact, dense spot in the centre, indicating a narrower and slower air stream from the nose. At higher flow rates (20 L/min), the aerosol formed a larger and more dispersed mark, suggesting a wider and more turbulent airflow that dispersed the particles over a larger area, despite the overall lower DPI, as more particles were retained at the front.

### 4.7. Descriptive Validation of Deposition Trends and DPI

Although we have not performed a one-to-one validation using identical nasal geometry and monodisperse reference aerosols, the qualitative trends observed in both regional particle deposition and the DPI are consistent with existing experimental and CFD evidence on nasal filtration of micron-sized particles.

The predominance of deposition in the anterior region of the nasal cavity and its shift towards these regions as inspiratory flow increases are consistent with a wide range of literature indicating that the nasal vestibule, nasal valve, and anterior nasal turbinates function as the primary filtration sites for micrometre-sized aerosols. In contrast, particle deposition in the posterior nasal cavity and nasopharynx becomes relatively minor at higher inspiratory flows [1,27,29,30,31,32,33,34].

For example, test results on a nasal model with 5.5 µm particles showed slight deposition in the nasopharynx at airflow rates above 10 L/min and strong anterior patterns at higher flows, confirming our observations of reduced deposition in the posterior regions as flow increases [29].

CFD simulations demonstrate that particle deposition sites shift toward the vestibule and anterior turbinates as particle size or flow rate increases, resulting in greater loading on the anterior turbinate surface [32]. Review-level evidence, synthesised from multiple CFD studies, indicates that higher flow rates produce more anterior hot spots, while smaller particles with lower inertia result in a more uniform distribution that is less concentrated in the anterior region [1].

CFD results for occupationally relevant wood dust demonstrate predominant deposition in the nasal valve and anterior nasal concha. Overall deposition increases with a higher proportion of coarse particles, whereas dusts with a greater fine particle fraction penetrate more deeply, which is directly consistent with our ranking of coarse and fine materials and anterior deposition load [30]. Similarly, experiments using models of the nasal cavity reveal that the anterior regions demonstrate the greatest sediment thickness across various flow rates, while smaller particles are distributed more uniformly and extend further toward the posterior [34].

In the case of unsteady breathing, the results of our ASL research fell between the constant 7.5 and 20 L/min conditions. This finding is consistent with computational evidence showing that cyclic breathing produces time-varying vortices and shear patterns. These patterns cannot be fully represented by a single steady-state simulation, even if the matched moment may appear similar [62].

The DPI measured in this study, defined as the increase in outlet filter mass relative to dispenser mass loss, serves as a penetration-type endpoint that can be compared at the trend level with the escape or penetration behaviour described in the literature [29,64,65]. Our results demonstrate that DPI decreased with increasing flow for all aerosols, with median values ranging from approximately 2–5% for coarse powders, such as wheat flour and wood dust, at high flow to approximately 12–20% for aerosols containing a higher proportion of fine particles, such as black carbon or Arizona Test Dust A3, at low or moderate flow. These findings are consistent with published data on nasal casts, which show that the fraction escaping downstream decreases sharply as nasal filtration increases, due to higher flow and greater particle inertia. For instance, for monodisperse particles with a size of 5.5 µm, total deposition efficiency in the nose increased significantly with flow measured at 6.2% at 10 L/min, 34.6% at 20 L/min, and 93.2% at 40 L/min, indicating a substantial reduction in downstream escape as flow increases [29]. CFD predictions for 10 µm particles similarly suggest that a significant proportion of particles may penetrate at lower flows, but penetration becomes negligible as the flow increases, with predicted total retention in the nasal cavity of 61%, 90%, and 100% at 7.5, 15, and 30 L/min, respectively [32].

Studies on occupational dust penetration and escape demonstrate a predictable hierarchy: very small particles show high escape rates, very large particles settle mainly in the anterior nasal region, and medium-sized particles (approximately 7 µm) display intermediate behaviour [64,65]. These datasets confirm that our observed DPI values, their dependence on flow, and the higher DPI values for aerosols with a greater proportion of fine particles compared to coarse powders are consistent with established penetration patterns in the region.

In the context of unsteady flow, our observation that particle deposition and DPI within the adult breathing profile fall between those in steady cases is consistent with previous reports indicating that cyclic inhalation can alter both the absolute amount and regional distribution of particle deposition. The same order is generally maintained based on particle inertia and size, with anterior deposition of inert particles remaining consistent [47,48,60,61]. Furthermore, published studies indicate that comparisons across investigations are most meaningful when focused on overall trends, since absolute particle deposition depends on the anatomical and physical characteristics of the replica. Consequently, the agreement described above is intended to be a descriptive validation of dominant trends rather than a strict quantitative validation [38,46].

### 4.8. Clinical Relevance and Prevention

The results of the study are clinically significant, as they provide insights into regional nasal deposition patterns that can inform targeted interventions to prevent and treat work-related respiratory diseases. The study shows that coarse occupational aerosols, wheat flour, and pine wood dust are mainly deposited in the anterior part of the nasal cavity, which can result in localised irritation, rhinitis, and nasal obstruction, while only a small amount reaches the nasopharynx. Fine-rich aerosols, such as Arizona Test Dust A3 and carbon black, penetrate deeper into the nasal passages, deposit more in the middle and posterior segments, and are more likely to reach the lower airways. Identifying particle deposition sites assists rhinoscopic and endoscopic examinations, guides provocation tests with workplace aerosols, supports the implementation of particle barrier protection measures, and shapes treatment strategies. Primary prevention should be based on reducing exposure at its source by using workplace controls, e.g., local exhaust ventilation, process enclosures, and the proper use of protective equipment (e.g., masks), especially during high-exposure tasks [3,9]. Secondary prevention aims to monitor possible symptoms, such as nasal obstruction, rhinorrhea, and work-related sneezing, to detect occupational rhinitis at an early stage and minimise its duration and severity [9]. As heavy physical work increases airflow and amplifies anterior nasal deposition of coarse particles while enabling higher downstream penetration of fine particles, workers should avoid strenuous tasks when dust levels are highest. Tertiary prevention includes targeted measures such as nasal saline rinsing and respiratory protection if exposure cannot be reduced. The results of the study may be relevant when interpreting nasal swab or rinse tests, planning sampling locations, and assessing which regions of the nasal cavity best reflect occupational aerosol exposure.

### 4.9. Limitations

The deposition patterns described here are representative of controlled laboratory conditions for the reference (“average”) adult nasal geometry under the tested inspiratory flow conditions. The results may provide mechanistic and comparative information for these reference airways. However, caution is needed when generalising to all anatomical variability and breathing behaviours. The 3D nasal cavity model does not capture the full spectrum of anatomical variability, and physiological changes such as the nasal cycle, turbinate enlargement, or mucociliary clearance cannot be simulated. The model is unilateral and constructed with rigid walls. It features a simplified mucus-like layer. Aerodynamic behaviour may be influenced by the hygroscopic properties of particles (such as flour), their cohesion, and agglomeration (carbon black and pine wood dust) relative to the measured particle size distribution. The potential effect of nasal hairs on filtration is neglected. DPI measurements may be affected by electrostatic effects and internal geometry and are presented only descriptively. In practice, occupational exposures may involve mixed contaminants, chronic exposure, and variable humidity and temperature, which are not considered here. Deposition metrics are based on surface coverage calculated from image analysis and may vary depending on the thresholding and segmentation parameters used. We measured only the pressure drop across the model, without reporting intranasal pressure loss distribution, which would require additional CFD simulations or spatially resolved measurements. Despite this, the experimental approach provides quantitative data on deposition patterns, complements CFD simulations, and enables visualisation and validation of simulated deposition results.

## 5. Conclusions

Experimental studies examining the deposition of occupationally relevant aerosols in a 3D model of the nasal cavity are scarce. The results confirm that the nose functions as an efficient filter for coarse workplace aerosols.

• Larger, broad-tailed aerosols, such as wheat flour and pine wood dust, having high Dv50 and containing few particles smaller than 10 µm, consistently deposit in the anterior nasal region, with little nasopharyngeal involvement. As a result, they have low downstream penetration, limiting exposure of the lower airways during nasal breathing.
• In contrast, aerosols with smaller Dv50 and more sub-10 µm particles, such as Arizona Test Dust A3 and carbon black, tend to deposit more in the middle and posterior nasal regions, with the anterior part of the nasal cavity still being the dominant deposition. These aerosols demonstrate elevated DPI at all flow rates, increasing the likelihood of reaching the tracheobronchial tree while still affecting the upper airway.
• The observed anterior shift (WA/SA↑; WP↓) with the increase in flow rate aligns with increased inertial impaction in the valve and turbinate region. Higher flow enhanced anterior filtration of coarse particles but increased deep-lung exposure to fine particles.
• With an unsteady breathing pattern, segmental deposition values fell between those at 7.5 and 20 L/min. Anterior segments aligned with peak inspiratory flow. Posterior segments corresponded to the longer mid-inspiratory phase. The ranking of aerosols remained unchanged.
• Downstream penetration (DPI) decreased with increasing flow for all materials, in the following order: Arizona Test Dust A3, carbon black, then wheat flour and pine wood dust.

These results provide insights into deposition behaviour, showing that factors such as particle size distribution, morphology, and flow rate influence how particles deposit by affecting their aerodynamics and Stokes number, especially for micron-sized particles.

The findings have important implications for assessing occupational exposure, developing intranasal interventions, and creating prevention strategies in occupational settings with high dust exposure.

## Figures and Tables

**Table 1:** Characterisation of occupationally relevant aerosols used in the study.

Material	Dv50 [µm]	Dv10–Dv90 [µm]	Span	%V < 10 µm
Wheat flour type 750	40.64	16.8–134.7	2.90	3.13
Pine wood sanding dust	43.35	15.52–116.3	2.325	4.31
Carbon black	23.43	5.39–84.79	3.389	22.88
Arizona Test Dust A3	15.40	3.32–44.83	2.695	36.44

**Table 2:** Pressure drop and nasal resistance of the unilateral nasal model.

Flow Rate [L/min]	Flow Rate [mL/s]	Δ*P* [Pa]	NR [Pa·s/mL]
5	83.3	27.9	0.335
7.5	125.0	62.9	0.503
20	333.3	446.9	1.341

**Table 3:** Segmental deposition on lateral wall and septum for four occupational aerosols. Values are reported as median and IQR (Q1–Q3) based on four independent repetitions per aerosol-flow condition (*n* = 4).

Air Flow	Location	Segment *	Wheat Flour 750	Pine Wood Dust	Carbon Black	Arizona Test Dust A3
			Median [%], IQR [Q1–Q3]			
5 L/min	Lateral wall	WA	56.2 [55.6–56.6]	50.9 [50.3–51.7]	40.9 [40.2–41.9]	37.9 [36.7–39.3]
		WM	15.1 [14.5–15.3]	15.3 [15.1–15.8]	16.8 [16.3–17.3]	17.6 [17.2–17.8]
		WP	6.0 [5.8–6.1]	6.2 [5.6–6.5]	8.2 [7.8–8.6]	9.2 [8.9–9.4]
	Septum	SA	57.9 [57.3–58.3]	50.5 [49.3–51.6]	45.9 [45.5–46.3]	41.9 [41.2–42.2]
		SP	12.0 [11.7–12.2]	12.5 [12.2–12.9]	17.0 [16.8–17.3]	15.7 [15.4–16.0]
7.5 L/min	Lateral wall	WA	64.0 [62.7–64.9]	57.3 [56.8–57.5]	49.9 [48.7–50.9]	44.0 [43.8–45.0]
		WM	15.6 [15.4–16.0]	16.1 [15.5–16.4]	18.3 [18.1–18.5]	18.0 [17.6–18.3]
		WP	5.3 [4.8–5.7]	5.1 [4.8–5.3]	7.5 [7.1–7.9]	8.4 [7.9–8.8]
	Septum	SA	62.1 [61.1–63.2]	55.7 [54.9–56.2]	48.9 [48.2–49.4]	45.1 [44.3–45.9]
		SP	13.2 [12.6–13.6]	12.1 [12.1–14.0]	16.3 [16.1–16.6]	14.8 [14.5–15.2]
20 L/min	Lateral wall	WA	70.0 [69.4–70.5]	65.8 [65.1–66.3]	56.2 [55.9–56.7]	57.8 [56.4–59.2]
		WM	17.0 [16.8–17.3]	16.7 [16.1–17.0]	18.9 [18.7–19.3]	19.3 [18.8–20.4]
		WP	4.3 [3.9–4.7]	4.1 [3.9–4.4]	5.9 [5.4–6.4]	6.2 [5.9–6.3]
	Septum	SA	67.0 [66.7–67.5]	62.3 [62.0–62.7]	50.6 [49.3–52.0]	51.0 [50.4–51.9]
		SP	15.4 [15.0–15.7]	13.9 [13.4–14.1]	18.4 [17.7–18.6]	16.8 [15.7–17.9]
unsteady	Lateral wall	WA	67.1 [66.7–67.6]	61.8 [60.4–63.2]	56.2 [55.6–56.6]	51.1 [50.5–52.1]
		WM	16.1 [15.8–16.5]	16.8 [16.4–16.9]	15.1 [14.5–15.3]	18.6 [17.5–19.7]
		WP	4.8 [4.2–5.2]	4.5 [4.2–4.8]	6.0 [5.8–6.1]	7.7 [7.4–7.9]
	Septum	SA	65.7 [65.1–66.1]	59.0 [58.0–60.1]	49.8 [48.6–50.8]	49.3 [47.5–50.8]
		SP	14.0 [13.7–14.2]	13.5 [12.7–14.5]	17.6 [16.9–17.9]	15.7 [15.5–16.3]

* Planned pairwise comparisons along the anterior–posterior axis (SA vs. SP; WA vs. WP; and WM vs. WP) were all statistically significant in the expected direction (more anterior > posterior; Wilcoxon rank-sum test, *p* < 0.02 (one-sided), *p* = 0.029 (two-sided)).

**Table 4:** Effect of airflow on segmental deposition on the nasal lateral wall and septum (one-way ANOVA).

Location	Segment	Wheat Flour 750	Pine Wood Dust	Carbon Black	Arizona Test Dust A3
		ANOVA (F(3,12), *p*, η^2^)			
Lateral wall	WA	83.16; *p* < 0.001; η^2^ = 0.954	48.57; *p* < 0.001; η^2^ = 0.924	48.54; *p* < 0.001; η^2^ = 0.924	87.49; *p* < 0.001; η^2^ = 0.956
	WP	5.05; *p* = 0.017; η^2^ = 0.558	6.07; *p* = 0.009; η^2^ = 0.603	7.31; *p* = 0.005; η^2^ = 0.646	27.23; *p* < 0.001; η^2^ = 0.872
Septum	SA	44.62; *p* < 0.001; η^2^ = 0.918	46.00; *p* < 0.001; η^2^ = 0.920	6.19; *p* = 0.009; η^2^ = 0.607	29.61; *p* < 0.001; η^2^ = 0.881
	SP	10.76; *p* = 0.001; η^2^ = 0.729	0.73; *p* = 0.553; η^2^ = 0.155	1.87; *p* = 0.188; η^2^ = 0.319	2.22; *p* = 0.139; η^2^ = 0.357

One-way ANOVA testing the effect of airflow (5 L/min, 7.5 L/min, 20 L/min, unsteady flow) on segmental deposition within anterior and posterior segments. Levene’s test assessed homogeneity of variances in all models (*p* > 0.20).

## Data Availability

The analysed datasets generated during the study are available from the corresponding author on reasonable request. The data are not publicly available due to data management and storage limitations.

## Supplementary Materials

The following supporting information can be downloaded at: https://www.mdpi.com/article/10.3390/bioengineering13020132/s1, Figure S1: Segmental deposition for four flow rates (5, 7.5, 20 L/min, unsteady) of carbon black on the lateral wall; Figure S2: Segmental deposition for four flow rates (5, 7.5, 20 L/min, unsteady) of wheat flour type 750 on the lateral wall; Figure S3: Segmental deposition for four flow rates (5, 7.5, 20 L/min, unsteady) of Arizona Test Dust A3 on the septum; Figure S4: Segmental deposition for four flow rates (5, 7.5, 20 L/min, unsteady) of pine wood dust on the septum; Table S1: Segment share of the total lateral wall and septal deposition for four occupational aerosols; Table S2. Downstream Penetration Index (DPI) (median, IQR) presented for four types of occupationally relevant aerosols and all flow rates used, together with their median particle diameter by volume (Dv50).

## Abbreviations and Nomenclature

The following nomenclature and abbreviations are used in this manuscript:

ANCM	adult nasal cavity
ANOVA	analysis of variance
AP	(vacuum) air pump
ASL	breathing simulator
CFD	Computational Fluid Dynamics
CT	computed tomography
DPI	Downstream Penetration Index, DPI = Δm_cassette/Δm_dispenser
Δm_cassette	mass gain of the outlet filter cassette
Δm_dispenser	mass loss from the powder dispenser
FBD	fluidised bed dispenser
HMW	high-molecular-weight
LMW	low-molecular-weight
IQR	interquartile range (Q1–Q3)
NC	nebuliser compressor
OA	occupational asthma
OR	occupational rhinitis
PSD	particle size distribution
Dv10	particle diameter at 10th percentile by volume
Dv50	median particle diameter by volume
Dv90	particle diameter at 90th percentile by volume
Span	(Dv90–Dv10)/Dv50
%V < 10 µm	volume fraction of particles with diameter < 10 µm
SA	septum anterior/anterior segment of the septum
SEM	scanning electron microscope
SP	septum posterior/posterior segment of the septum
STL	stereolithography
WA	wall anterior/anterior segment of the lateral wall
WM	wall middle/middle segment of the lateral wall
WP	wall posterior/posterior segment of the lateral wall (nasopharynx region)
WRR	work-related rhinitis
3D	three-dimensional
*Q*	volumetric inhalation flow rate (per nostril, unless stated otherwise)
VT	tidal volume
I:E	inspiration-to-expiration ratio
TI	inspiratory time
TE	expiratory time
URC	ASL 5000 profile parameter (device setting)
R in/R out	inspiratory/expiratory resistance setting (ASL 5000)
C	compliance setting (ASL 5000)
P max	maximum pressure/effort setting (ASL 5000)
Stk	Stokes number
*u*	characteristic flow (particle) velocity
*ρ*	particle density
*d*	effective particle diameter
*μ*	dynamic viscosity of air
*L*	characteristic airway length/size
*p*	*p*-value
α	significance level
η ^2^	effect size (eta-squared)
*F (df*_1_*, df*_2_*)*	ANOVA F statistic with degrees of freedom
*W*	Wilcoxon rank-sum test statistic
t	Welch’s *t*-test statistic
χ^2^	Kruskal–Wallis test statistic
*A*	(cross-sectional) area
*P*	perimeter
D_h_	hydraulic diameter
mCSA	minimal cross-sectional area
NR	nasal resistance
*ΔP* IP	pressure drop impaction parameter
SD	standard deviation
MANOVA	multivariate analysis of variance
HSD	Tukey’s Honestly Significant Difference
PCA PIF	principal component analysis peak inspiratory flow
