# Supplementary material for: Deposition of Occupational Aerosol Particles in a Three-Dimensional Adult Nasal Cavity Model: An Experimental Study
Source: Bioengineering (Basel). 2026 Jan 23;13(2):132. doi: 10.3390/bioengineering13020132 (PMC12938197; doi:10.3390/bioengineering13020132)
Supplement: Supplementary file 1 [file bioengineering-13-00132-s001.zip › supplementary figures_captions .pdf]

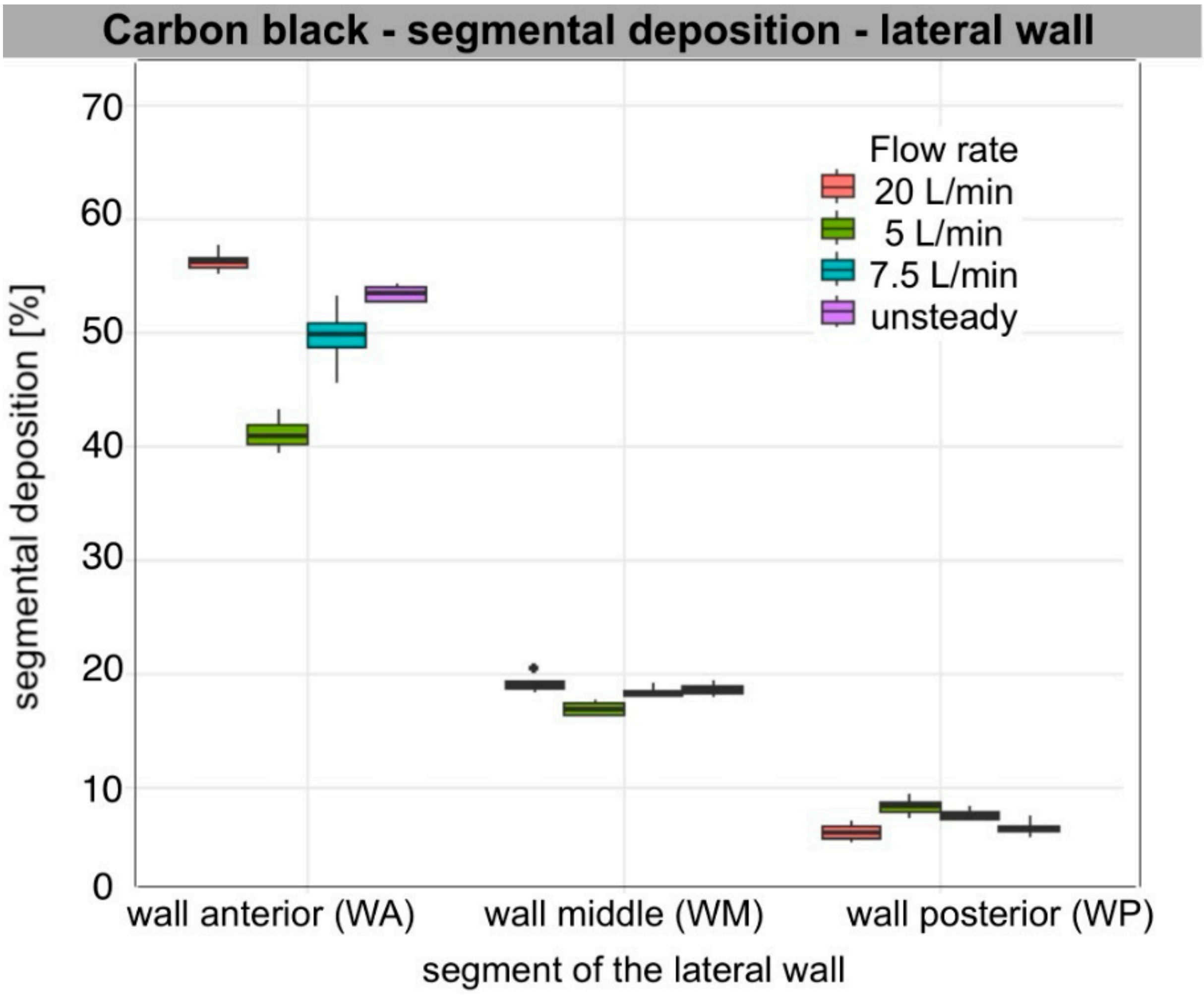

Supplementary Figure S1. Segmental deposition for four flow rates (5, 7.5, 20L/min, unsteady) of carbon black on the lateral wall.

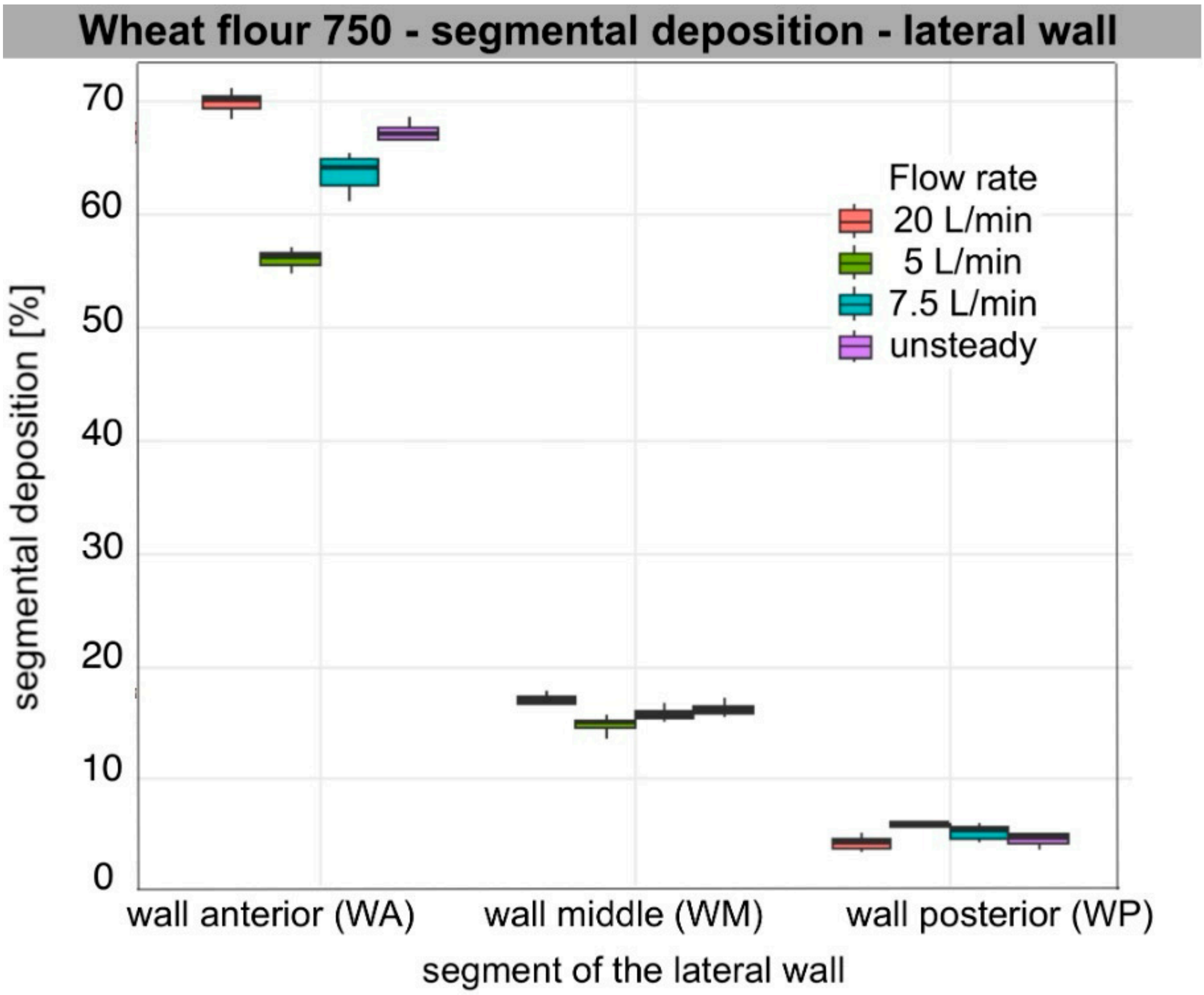

Supplementary Figure S2. Segmental deposition for four flow rates (5, 7.5, 20L/min, unsteady) of wheat flour type 750 on the lateral wall.

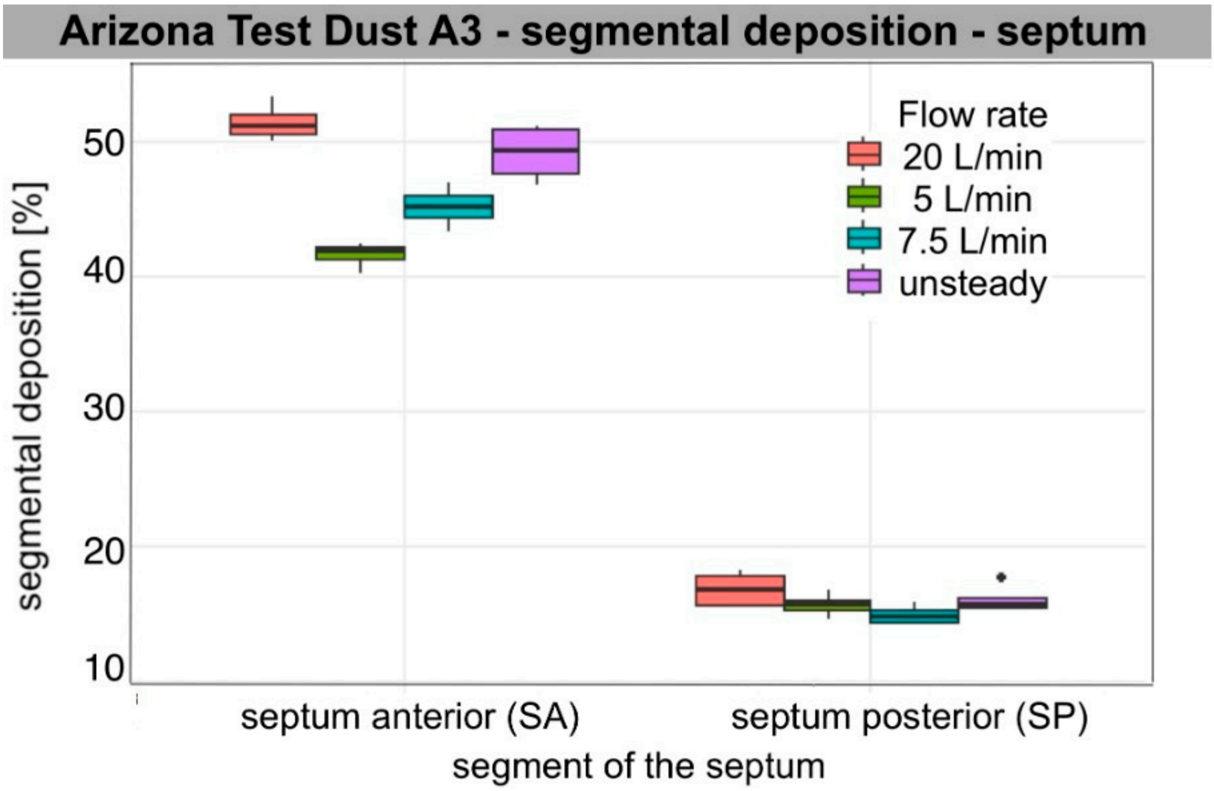

Supplementary Figure S3. Segmental deposition for four flow rates (5, 7.5, 20L/min, unsteady) of Arizona Test Dust A3 on the septum.

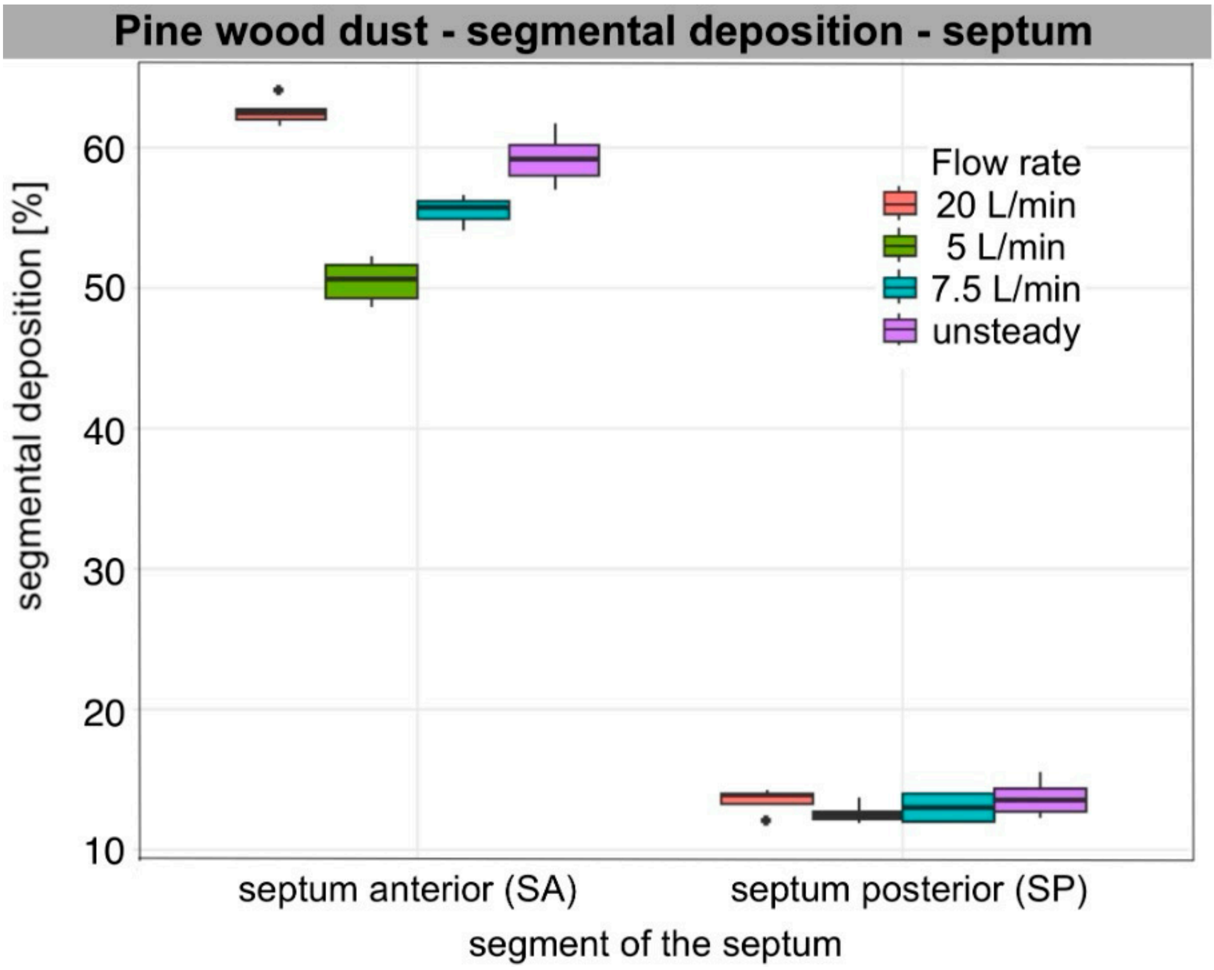

Supplementary Figure S4. Segmental deposition for four flow rates (5, 7.5, 20L/min, unsteady) of. pine wood dust on the septum.
