# Supplementary material for: Deposition of Occupational Aerosol Particles in a Three-Dimensional Adult Nasal Cavity Model: An Experimental Study
Source: Bioengineering (Basel). 2026 Jan 23;13(2):132. doi: 10.3390/bioengineering13020132 (PMC12938197; doi:10.3390/bioengineering13020132)
Supplement: Supplementary file 1 [file bioengineering-13-00132-s001.zip › supplementary table 1 and table 2 .pdf]

Supplementary Table S1. Segment share of the total lateral wall and septal deposition for four occupational aerosols.

Air flow	Location	Segment	Wheat flour 750	Pine wood dust	Carbon black	Arizona Test Dust A3
			Median [%], IQR [Q1-Q3]			
5L/min	Lateral wall	WA	10.3[10.2-10.4]	9.3[9.2-9.5]	7.5[7.3-7.7]	6.9[6.7-7.2]
		WM	9.9[9.5-10.1]	10.0[9.9-10.4]	11.0[10.7-11.3]	11.5[11.3-11.7]
		WP	1.0[0.9-1.0]	1.0[0.9-1.0]	1.3[1.2-1.4]	1.5[1.4-1.5]
	Septum	SA	12.6[12.5-12.7]	11.0[10.7-11.2]	10.2[10.1-10.3]	9.1[9.0-9.2]
		SP	9.4[9.1-9.6]	9.7[9.5-10.1]	12.2[13.1-13.5]	12.3[12.0-12.5]
7.5L/min	Lateral wall	WA	11.7[11.5-11.9]	10.5[10.4-10.5]	9.3[8.9-9.4]	8.1[8.0-8.2]
		WM	10.2[10.1-10.5]	10.5[10.3-10.7]	12.0[11.9-12.2]	11.8[11.6-12.0]
		WP	0.8[0.8-0.9]	0.8[0.76-9.85]	1.2[1.1-1.3]	1.4[1.3-1.4]
	Septum	SA	13.5[13.3-13.8]	12.1[12.0-12.2]	10.7[10.5-10.8]	9.8[9.6-10.0]
		SP	10.3[9.8-10.6]	10.2[9.4-10.9]	12.7[12.6-13.0]	11.5[11.3-11.9]
20L/min	Lateral wall	WA	12.8[12.7-12.9]	12.0[11.9-12.1]	10.0[9.9-10.1]	10.6[10.3-10.8]
		WM	11.2[11.0-11.3]	10.9[10.6-11.2]	12.4[12.3-13.2]	12.7[12.3-13.4]
		WP	0.7[0.6-0.7]	0.7[0.6-0.7]	1.1[1.0-1.1]	1.0[0.9-1.0]
	Septum	SA	14.6[14.5-14.7]	13.6[13.5-13.7]	11.0[10.7-11.3]	11.1[11.0-11.3]
		SP	12.0[11.7-12.3]	10.9[10.4-11.0]	13.6[13.2-14.1]	13.1[12.3-14.0]
unsteady	Lateral wall	WA	12.3[12.2-12.4]	11.3[11.1-11.6]	9.6[9.5-9.7]	9.3[9.2-9.5]
		WM	10.6[10.4-10.8]	11.0[10.7-11.1]	12.2[12.0-12.5]	12.2[11.5-13.0]
		WP	0.8[0.7-0.8]	0.7[0.7-0.8]	1.2[1.1-1.2]	1.2[1.2-1.3]
	Septum	SA	14.3[14.2-14.4]	12.9[12.6-13.1]	10.8[10.5-11.1]	10.7[10.4-11.1]
		SP	10.9[10.7-11.1]	10.6[10.0-11.3]	13.1[12.5-13.6]	12.2[12.1-12.7]

Supplementary Table S2. Downstream Penetration Index (DPI) (median, IQR) presented for four types of occupationally relevant aerosols and all flow rates used, together with their median particle diameter by volume (Dv50).

Aerosol	Dv50 [µm]	Flow rate	DPI (median, IQR [Q1-Q3])
Wheat flour 750	40.64	5 L/min	4.0 [2.0-7.0]
		7.5 L/min	3.5 [2.0-6.0]
		20 L/min	2.0 [1.0-3.6]
		unsteady	2.5 [1.5-4.0]
Pine wood dust	43.35	5 L/min	5.0 [3.0-9.0]
		7.5 L/min	4.0 [2.5-7.0]
		20 L/min	2.0 [1.0-4.0]
		unsteady	3.0 [1.5-5.0]
Carbon black	23.43	5 L/min	15.0 [10.0-22.0]
		7.5 L/min	12.0 [8.0-18.0]
		20 L/min	7.0 [4.0-11.0]
		unsteady	9.0 [6.0-14.0]
Arizona Test Dust A3	15.40	5 L/min	20.0 [14.0-30.0]
		7.5 L/min	17.0 [12.0-25.0]
		20 L/min	10.0 [7.0-15.0]
		unsteady	13.0 [9.0-19.0]
