# Supplementary figures and images for: Deposition of Occupational Aerosol Particles in a Three-Dimensional Adult Nasal Cavity Model: An Experimental Study
Source: Bioengineering (Basel). 2026 Jan 23;13(2):132. doi: 10.3390/bioengineering13020132 (PMC12938197; doi:10.3390/bioengineering13020132)

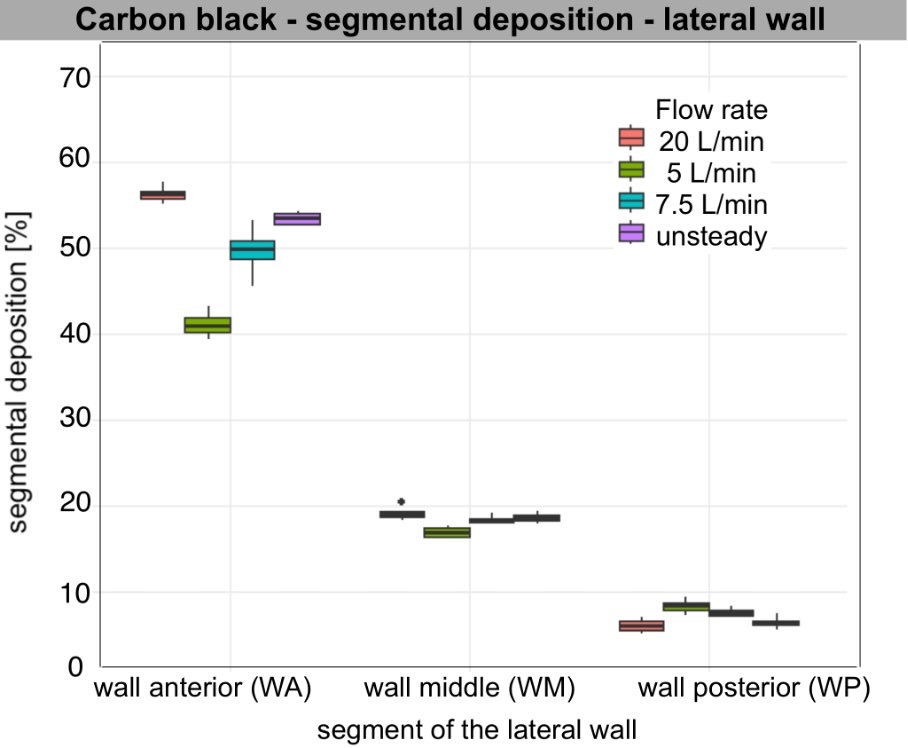

Supplement: Supplementary file 1 [file bioengineering-13-00132-s001.zip › S1 Supplementary figure S1.jpg]

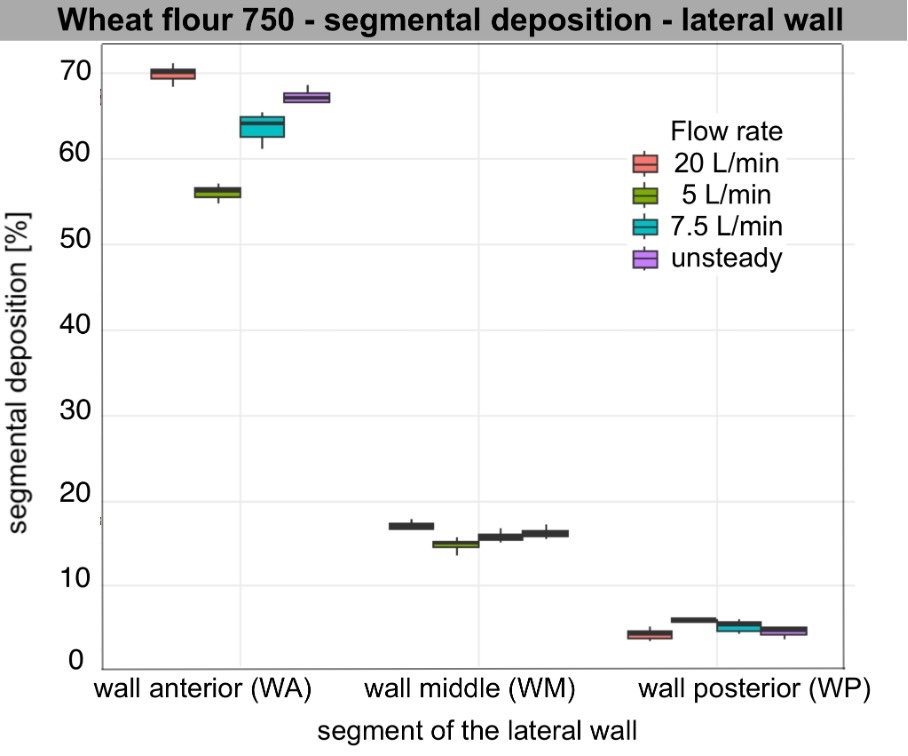

Supplement: Supplementary file 1 [file bioengineering-13-00132-s001.zip › S2 Supplementary figure S2.jpg]

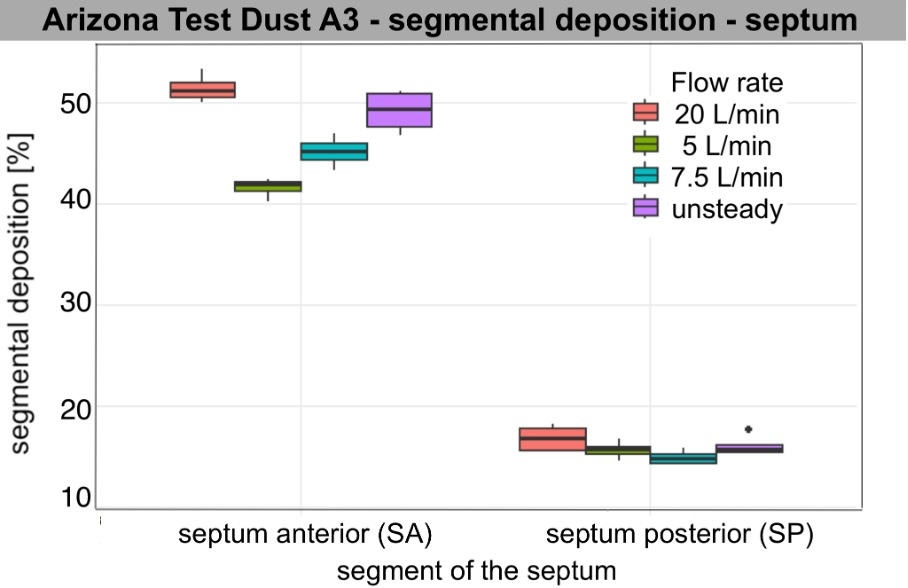

Supplement: Supplementary file 1 [file bioengineering-13-00132-s001.zip › S3 Supplementary figure S3.jpg]

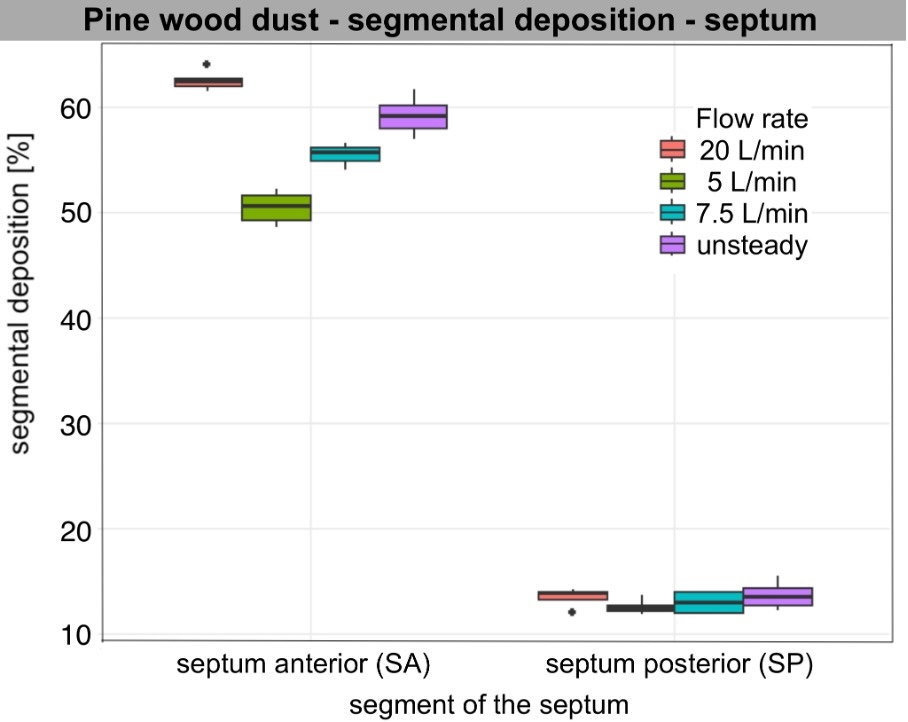

Supplement: Supplementary file 1 [file bioengineering-13-00132-s001.zip › S4 Supplementary figure S4.jpg]
